# Different polarization and functionality of CD4+ T helper subsets in people with post-COVID condition

**DOI:** 10.3389/fimmu.2024.1431411

**Published:** 2024-08-27

**Authors:** Clara Sánchez-Menéndez, Olivia de la Calle-Jiménez, Elena Mateos, Lorena Vigón, Daniel Fuertes, María Aranzazu Murciano Antón, Esther San José, Valentín García-Gutiérrez, Miguel Cervero, Montserrat Torres, Mayte Coiras

**Affiliations:** ^1^ Immunopathology and Viral Reservoir Unit, National Center of Microbiology, Instituto de Salud Carlos III, Madrid, Spain; ^2^ PhD Program in Biomedical Sciences and Public Health, Universidad Nacional de Educación a Distancia (UNED), Madrid, Spain; ^3^ Hematology and Hemotherapy Service, Instituto Ramón y Cajal de Investigación Sanitaria (IRYCIS), Hospital Universitario Ramón y Cajal, Madrid, Spain; ^4^ Biomedical Research Center Network in Infectious Diseases (CIBERINFEC), Instituto de Salud Carlos III, Madrid, Spain; ^5^ Internal Medicine Service, Hospital Universitario Clínico San Carlos, Madrid, Spain; ^6^ AIDS Immunopathology, National Center of Microbiology, Instituto de Salud Carlos III, Madrid, Spain; ^7^ School of Telecommunications Engineering, Universidad Politécnica de Madrid, Madrid, Spain; ^8^ Family Medicine, Centro de Salud Doctor Pedro Laín Entralgo, Alcorcón, Madrid, Spain; ^9^ International PhD School, Universidad Rey Juan Carlos, Alcorcón, Madrid, Spain; ^10^ Immunomodulation Unit, Department of Health Sciences, Faculty of Biomedical and Health Sciences, European University of Madrid, Madrid, Spain; ^11^ School of Medicine, Universidad Alfonso X El Sabio, Madrid, Spain

**Keywords:** post-covid condition, CD4+ T cells, T helper polarization, cytokines, Th1, Th2, Th17

## Abstract

**Introduction:**

After mild COVID-19 that does not require hospitalization, some individuals develop persistent symptoms that may worsen over time, producing a multisystemic condition termed Post-COVID condition (PCC). Among other disorders, PCC is characterized by persistent changes in the immune system that may not be solved several months after COVID-19 diagnosis.

**Methods:**

People with PCC were recruited to determine the distribution and functionality of CD4+ T helper (Th) subsets in comparison with individuals with mild, severe, and critical presentations of acute COVID-19 to evaluate their contribution as risk or protective factors for PCC.

**Results:**

People with PCC showed low levels of Th1 cells, similar to individuals with severe and critical COVID-19, although these cells presented a higher capacity to express IFNγ in response to stimulation. Th2/Th1 correlation was negative in individuals with acute forms of COVID-19, but there was no significant Th2/Th1 correlation in people with PCC. Th2 cells from people with PCC presented high capacity to express IL-4 and IL-13, which are related to low ventilation and death associated with COVID-19. Levels of proinflammatory Th9 and Th17 subsets were significantly higher in people with PCC in comparison with acute COVID-19, being Th1/Th9 correlation negative in these individuals, which probably contributed to a more pro-inflammatory than antiviral scenario. Th17 cells from approximately 50% of individuals with PCC had no capacity to express IL-17A and IL-22, similar to individuals with critical COVID-19, which would prevent clearing extracellular pathogens. Th2/Th17 correlation was positive in people with PCC, which in the absence of negative Th1/Th2 correlation could also contribute to the proinflammatory state. Finally, Th22 cells from most individuals with PCC had no capacity to express IL-13 or IL-22, which could increase tendency to reinfections due to impaired epithelial regeneration.

**Discussion:**

People with PCC showed skewed polarization of CD4+ Th subsets with altered functionality that was more similar to individuals with severe and critical presentations of acute COVID-19 than to people who fully recovered from mild disease. New strategies aimed at reprogramming the immune response and redirecting CD4+ Th cell polarization may be necessary to reduce the proinflammatory environment characteristic of PCC.

## Introduction

Infection by SARS-CoV-2 can lead to different presentations of Coronavirus disease 2019 (COVID-19), ranging from asymptomatic infections to critical forms that require hospitalization in the intensive care unit (ICU) (1). Advanced age and male gender are determining factors in the development of disease severity, as well as the existence of comorbidities such as diabetes mellitus, hypertension, obesity, and cancer (2, 3). Even though global vaccination against COVID-19 was rapidly implemented, this disease may still be severe or critical due to new emerging viral variants and waning vaccine protection, as well as in unvaccinated or immunocompromised people (4).

Symptoms related to COVID-19 normally disappear in the first 4 weeks after infection, although in some cases they may persist up to 12 weeks (5, 6). However, there is a meaningful percentage of the population that may develop long-term symptoms and complications related to the viral infection. This post-viral syndrome is referred to as Long COVID, Long-haul COVID, or Post COVID-19 condition (PCC). According to the World Health Organization (WHO), the clinical case definition of PCC is the persistence of COVID-19 symptoms for at least two months in individuals with a history of probable or confirmed SARS-CoV-2 infection, usually three months from the onset of COVID-19, and that cannot be explained by an alternative diagnosis (6). The incidence of PCC within the population has been roughly estimated as 10% after SARS-CoV-2 infection (7), not only in people who needed hospitalization during the acute infection but also in individuals who had mild forms of COVID-19 (8, 9). The most important risk factors identified in PCC are female gender, non-white ethnicity, socioeconomic deprivation, smoking history (former or current), obesity, pre-existing medical conditions, and being unvaccinated against COVID-19 before first SARS-CoV-2 infection (10, 11). However, some cases of PCC have also been described as a consequence of receiving vaccination against SARS-CoV-2 infection (12, 13). Interestingly, the fact that women appear to be more predisposed to develop PCC, opposite to the trend observed in acute COVID-19, seems to bear a resemblance to autoimmune-like processes (14). Therefore, several hypotheses are behind the emergence of PCC, including chronic inflammation induced by viral persistence, sustained immune deregulation, or hypersensitivity (15).

While conventional SARS-CoV-2 infection mainly results in respiratory disorders, such as cough, dyspnea, bronchial hyperreactivity, or pneumonia, PCC presents both multifactorial origins and symptomatology (5, 14, 16). Organ damage can be found in multiple systems, with emphasis on neurological symptoms such as memory loss, brain fog, migraine, or dysautonomia (16). Chronic fatigue and post-exertional malaise are also prevalent symptoms that can lead to lifelong impairment (4, 11). These symptoms are thought to be a consequence of the uncontrolled inflammatory response induced by SARS-CoV-2, which can sometimes persist to become chronic inflammation (17). This exacerbated inflammatory response is directly related to worse clinical outcomes during acute COVID-19. In these cases, the immune system is deregulated and an overproduction of proinflammatory cytokines occurs, known as the “cytokine storm”, leading to organ damage and death (18). In addition to this hyperinflammatory response, high levels of cytotoxic cells such as Natural Killer (NK) have been observed in critical patients, although these cells usually express exhaustion markers such as programmed cell death protein 1 (PD-1) on their surface (19), and exhibit low cytotoxic and cytokine production capabilities (20). Similarly, the levels of CD8+ T lymphocytes are maintained in critical patients but their cytotoxic activity is greatly reduced, resulting in a deficient virus clearance that may severely affect the outcome of the disease (20).

CD4+ lymphopenia often occurs during severe and critical COVID-19, likely due to the immune exhaustion and the high concentration of chemokines that inhibit hematopoietic progenitors such as IP-10 or MCP-1 (21). Moreover, a deficient antigen presentation for T cells contributes to the impaired and inefficient antiviral immune response during COVID-19 (22). Antigen presentation is essential for the development of the most appropriate type of immune response for each situation as it induces CD4+ T helper (Th) cell polarization in combination with other multiple factors like the cytokine microenvironment (23). CD4+ Th1 cells mainly mediate the elimination of intracellular pathogens such as viruses through the production of IFNγ and IL-2, while CD4+ Th2 cells primarily participate in extracellular pathogen clearance and immunoglobin class-switching through the production of multiple cytokines like IL-4, IL-5, IL-10, and IL-13 (23, 24). On the other hand, CD4+ Th9 cells are involved in hypersensitivity and allergic reactions, as well as helminth infections, mostly secreting IL-9 (25), while CD4+ Th17 cells are generally found in digestive and respiratory mucous membranes where they release cytokines such as IL-17A, IL-17F, IL-22, and IL-6 that help establish a pro-inflammatory microenvironment which deregulation has been linked to autoimmune diseases such as rheumatoid arthritis, systemic lupus erythematosus, or asthma (26). Finally, CD4+ Th22 cells stimulate keratinocyte proliferation through the release of IL-13 and IL-22, and participate in endothelial repair but also in inflammatory skin conditions like psoriasis and Crohn´s disease (27). Therefore, imbalanced CD4+ Th polarization may be responsible for the development of different diseases as a consequence of the deregulated immune response. In fact, an imbalance in the ratio Th1/Th2 has been associated with a poor outcome of acute COVID-19 (28). As occurs in other viral diseases, a strong Th1 response is indicative of good disease progression, while a predominant Th2 response evolves to worse clinical outcomes (29, 30). However, while most of the immune parameters that are deregulated during acute COVID-19 become normal after the resolution of the infection, people with PCC show persistently exhausted T cells with reduced memory subsets and increased IFNγ production (7, 16).

The changes that are produced in the immune response during different presentations of COVID-19 have been widely studied (3, 19, 20, 29–31), as well as during PCC (11, 17, 32, 33). However, the cause for the impaired immune response induced by SARS-CoV-2 infection that persists in PCC has not been fully determined. Due to the central role of CD4+ Th cell polarization in the development of an adequate response against infectious agents, in this study we analyzed the levels and functionality of CD4+ Th cell populations in a cohort of people with PCC, in comparison with three cohorts of individuals with mild, severe, and critical presentations of acute COVID-19. The results could contribute to advance towards a better understanding of the mechanisms underlying PCC and the design of new effective therapeutic strategies.

## Materials and methods

### Study subjects

A total of 79 individuals with different clinical presentations of COVID-19 were recruited for this study in Madrid (Spain) between April 2020 and March 2021. Sixty participants were classified into three cohorts according to disease severity during COVID-19, following the guidelines of the World Health Organization (WHO) (1): Mild COVID-19 (n=20), SARS-CoV-2 infection without hypoxia or pneumonia symptoms in which no hospitalization was required for recovery and symptoms subside in 4-12 weeks; Severe COVID-19 (n=20), SARS-CoV-2 infection with pneumonia symptoms (cough, fever, dyspnea) and one of the following conditions: respiratory rate with more than 30 breaths per minute, severe respiratory distress, or oxygen saturation <90%, and they tend to require inpatient care; and Critical COVID-19 (n=20), SARS-CoV-2 infection that required admission to the ICU due to acute respiratory distress syndrome (ARDS), bilateral pneumonia, pulmonary infiltrates, oxygenation impairment, sepsis, septic shock, or acute thrombosis. A fourth cohort was formed by 19 participants diagnosed with PCC by their Primary Care physician. According to WHO, PCC is defined by the experience of a range of symptoms (i.e. fatigue, muscle or joint pain, breathlessness, impaired sleep, depression and anxiety, loss of smell and taste, headache, and “brain fog”, defined as difficulty in thinking or concentrating (34–36)) usually 3 months after the onset of COVID-19, that last for at least 2 months and cannot be explained by an alternative diagnosis (6). Sample size was calculated using the sample size calculator Granmo (37) based on a level of confidence of 95% (α=0.05) and power of the analysis of 100% (β=0.2).

Participants with mild COVID-19, as well as those with PCC, were recruited at the Primary Healthcare Center Doctor Pedro Laín Entralgo (Madrid, Spain) and through the Spanish Long COVID Patient´s Association (AMACOP) and Long COVID ACTS (Autonomous Communities Together Spain) Association. Participants with severe and critical COVID-19 were recruited at Hospital Universitario Ramón y Cajal (Madrid, Spain) during hospitalization. Inclusion criteria were being over 18 years old, having at least one positive RT-qPCR assay for SARS-CoV-2 in nasopharyngeal smear, and fulfilling WHO criteria to be included in one of the four cohorts.

### Ethical statement

All individuals gave informed written consent to participate in the study before donating one blood sample. Protocols for this study (CEI PI 32_2020-v2; CEI PI 72_2022) were prepared in accordance with the Helsinki Declaration and previously reviewed and approved by the Ethics Committee of Instituto de Salud Carlos III (IRB IORG0006384) and the Commission of the Care Management of Primary Care of the Comunidad de Madrid (Spain). Current Spanish and European Data Protection Acts secured the confidentiality and anonymity of all participants.

### Blood samples processing

Blood samples were collected in EDTA Vacutainer tubes (Becton Dickinson, Madrid, Spain). Peripheral blood mononuclear cells (PBMCs) and plasma were immediately isolated from whole blood by centrifugation in a Ficoll-Hypaque density gradient (Corning, NY, USA). PBMCs were cryopreserved and stored in liquid nitrogen until the moment of analysis.

### Phenotyping of CD4+ Th cell populations

PBMCs were stained with conjugated antibodies CD3-PE (Immunostep, Salamanca, Spain) and CD8-APC-H7 (BD Biosciences, San Jose, CA). CD3+CD8- were assumed to be CD4+ T cells to include those cells with deregulated CD4 expression caused by SARS-CoV-2 infection (38). Cells were also stained with CXCR3-BV421, CCR4-PECy7, CCR6-BV650 and CCR10-BUV395 (BD Biosciences) to phenotypically characterize and quantify CD4+ Th cell subpopulations as follows: Th1 (CXCR3+CCR6-), Th2 (CCR4+CCR6-), Th17 (CCR4+CCR6+), Th9 (CCR6+CCR4-), and Th22 (CCR4+CCR6+CCR10+). Data acquisition was performed in LSRFortessa X-20 flow cytometer with FACS Diva Software (BD Biosciences) and data was analyzed with Flow-Jo_V10.8.1 (Treestar). The gating strategy for the phenotyping of CD4+ Th cell populations is shown in **Supplementary Figure 1**.

### Cytokine expression by CD4+ Th cell populations

The capacity to express representative cytokines by each CD4+ Th cell subpopulation was measured by flow cytometry after stimulation with phorbol 12-myristate 13-acetate (PMA) (25ng/ml) and ionomycin (1.5µg/ml) for 4h at 37°C in the presence of brefeldin A (BD GolgiPlug, BD Biosciences) that blocks the anterograde exocytotic transport through the Golgi complex (39). After cell surface staining of each Th cell subset, cells were fixed and permeabilized with IntraPrep Permeabilization reagent (Immunostep) and intracellularly stained with the following antibodies: IFNγ-FITC (Beckman Coulter, Brea, CA), IL-4-APC, IL-9-PercP, IL-13-BV711, IL-17A-BV510, and IL-22-AF647 (BD Biosciences). Data acquisition was performed with LSRFortessa X-20 flow cytometer (BD Biosciences), and data was analyzed using FACS Diva Software (BD Biosciences) and Flow-Jo_V10.8.1 (Treestar). The gating strategy for the intracellular staining of cytokines expressed by each CD4+ Th cell subset is shown in **Supplementary Figure 2**.

### Statistical analysis

Statistical analysis was performed with GraphPad Prism v10.2.1 (GraphPad Software Inc.) and STATA 14.2 software (StataCorp LLC, College Station, TX). Quantitative variables were described as the median and interquartile range (IQR) and qualitative variables as absolute or relative frequencies. Samples’ normal distribution was determined using Shapiro-Wilk test. Significance between data of different cohorts was determined with ordinary one-way Analysis of Variance (ANOVA) and Tukey post-test or with Kruskal-Wallis test and Dunn’s multiple comparisons test, depending on data normality. Qualitative data were compared by Fisher´s exact test or chi-square test, as appropriate. Simple and logistic regressions were applied to estimate the odds ratio (OR) and 95% confidence interval (CI) for associations between the levels of Th subsets and the expression of related cytokines with the development of critical, severe, or persistent forms of COVID-19 in comparison with participants with mild COVID-19. To analyze data correlation and compute the Pearson coefficient between all Th subsets per cohort, we employed a combination of Python libraries, including Scikit-Learn (40) and Pandas (41, 42) libraries. For the generation of regression plots, the Seaborn library (43) was used. P-values (p) < 0.05 were considered statistically significant in all comparisons.

## Results

### Clinical and sociodemographic characteristics of participants

For this study, 79 individuals who had SARS-CoV-2 infection confirmed by positive RT-qPCR in nasopharyngeal smear or positive serology for IgM were recruited and divided into different cohorts depending on the severity of the infection and according to WHO classification (1): Mild (n=20), Severe (n=20), and Critical (n=20) COVID-19, and PCC (n=19). Main demographic and clinical characteristics of these cohorts are summarized in **Table 1** and detailed in **Supplementary Table 1**. Participants with Mild, Severe, and Critical COVID-19 were evenly divided into males (50%) and females (50%), while the PCC cohort was comprised mostly of females (95%), as female gender is one factor associated with higher risk of developing this syndrome (44). Median age at infection was 43 years old (interquartile range (IQR) 28-59) for the Mild group, 50 years old (IQR 44-55) for the Severe group, 53 years old (IQR 47-59) for the Critical group, and 42 years old (IQR 37-46) for the PCC cohort. None of the participants were vaccinated against COVID-19 before being infected with SARS-CoV-2, as vaccination began in Spain in December 2020 and there was no vaccine available for non-risk groups at the time of sample collection. Median length of hospital stay (LOS) for individuals with Severe and Critical COVID-19 was 7 (IQR 6-11) and 45 days (IQR 28-80), respectively, while participants from Mild and PCC cohorts were not hospitalized during acute COVID-19. Only participants from Critical cohort were admitted to the ICU for a median stay of 18 days (IQR 8-40).

**Table 1:** Sociodemographic and clinical data of all participants in the study.

	Acute infection			PCC n=19	p value		
	Mild COVID-19 (Recovered) n=20	Severe COVID-19 n=20	Critical COVID-19 n=20		Mild COVID-19 *versus* PCC	Severe COVID-19 *versus* PCC	Critical COVID-19 *versus* PCC
Age at infection, years; median (IQR)	43 (28–59)	50 (44–55)	53 (47–59)	42 (37–46)	0.6165	0.1662	**0.0004**
Gender male/female; n (%)	10/10 (50/50)	10/10 (50/50)	10/10 (50/50)	1/18 (5/95)	**0.0033**	**0.0033**	**0.0033**
Days from clinical onset to sample, median (IQR)	85 (80–95)	13 (9–17)	25 (12–35)	330 (342–352)	**<0.0001**	**<0.0001**	**<0.0001**
Hospitalization due to COVID-19							
LOS, days; median (IQR)	0 (0)	7 (6–11)	45 (28–80)	0 (0)	1	**<0.0001**	**<0.0001**
ICU stay, days; median (IQR)	0 (0)	0 (0)	18 (8–40)	0 (0)	1	1	**<0.0001**
Symptoms during acute COVID-19 for mild, severe, and critical participants; persistent symptoms in participants with PCC							
Cough and/or expectoration; n (%)	11 (55)	9 (45)	13 (65)	14 (75)	0.3203	0.1053	0.7311
Dyspnea: n (%)	3 (15)	11 (55)	14 (70)	10 (53)	**0.0187**	1	0.3332
Fever; n (%)	12 (60)	16 (80)	15 (75)	12 (63)	1	0.3008	0.5006
Pneumonia; n (%)	1 (5)	17 (85)	19 (95)	6 (32)	**0.0436**	**0.0011**	**<0.0001**
Lethargy; n (%)	1 (5)	2 (10)	6 (30)	17 (89)	**<0.0001**	**<0.0001**	**0.0002**
Asthenia; n (%)	12 (60)	3 (15)	6 (30)	18 (95)	**0.0197**	**<0.0001**	**<0.0001**
Memory loss; n (%)	0 (0)	0 (0)	0 (0)	16 (84)	**<0.0001**	**<0.0001**	**<0.0001**
Arrhythmia; n (%)	0 (0)	0 (0)	0 (0)	12 (63)	**<0.0001**	**<0.0001**	**<0.0001**
Palpitations; n (%)	0 (0)	0 (0)	0 (0)	6 (32)	**0.0202**	**0.0202**	**0.0202**
Comorbidities							
Diabetes mellitus; n (%)	1 (5)	3 (15)	4 (20)	1 (5)	1	0.6050	0.3416
Dyslipidemia; n (%)	3 (15)	5 (25)	4 (20)	4 (21)	0.6948	1	1
Arterial hypertension; n (%)	4 (20)	6 (30)	7 (23)	2 (11)	0.6614	0.2351	0.1274
Treatment during acute COVID-19 for mild, severe, and critical participants; current treatment in participants with PCC							
Antibiotics	0 (0)	6 (30)	14 (70)	12 (63)	**<0.0001**	**0.0562**	0.7411
Anticoagulants	0 (0)	6 (30)	6 (30)	6 (32)	**0.0083**	1	1
Immunomodulators	1 (5)	15 (75)	16 (80)	5 (26)	0.0915	**0.0038**	**0.0012**
Antivirals	0 (0)	1 (5)	7 (35)	8 (42)	**0.0012**	**0.0084**	0.7475
Oxygen therapy	0 (0)	5 (25)	12 (60)	1 (11)	0.4872	0.1818	**0.0004**
Invasive mechanical ventilation	0 (0)	0 (0)	15 (75)	0 (0)	1	1	**<0.0001**
Exitus	0 (0)	0 (0)	4 (20)	0 (0)	1	1	0.1060

ICU, Intensive care unit; IQR, Interquartile range; LOS, Length of Hospital Stay; NSAIDs, Non-Steroidal Anti-Inflammatory Drugs; PCC, Post-COVID condition.

Significant p-values are indicated in bold letters.

General symptoms of acute COVID-19 such as cough and expectoration, dyspnea, and fever were reported in all cohorts with varying degrees of frequency. Of them, dyspnea was reported during acute infection in 15%, 55%, and 70% of Mild, Severe, Critical participants, respectively, and in 53% of PCC participants as a persistent symptom. Fever was reported by participants from all groups, but no significant differences were observed between cohorts. Pneumonia was reported in 5%, 85%, 95%, and 32% of Mild, Severe, Critical, and PCC participants, respectively, during acute infection. Other symptoms more closely associated with PCC than with acute COVID-19 were also compiled: 89% and 95% of PCC participants reported persistent lethargy and asthenia, respectively, in comparison with 5%, 10%, and 30% of Mild, Severe and Critical participants who reported lethargy, and 60%, 15%, and 30%, respectively, who reported asthenia during the acute infection. Other symptoms such as persistent memory loss (84%), arrhythmia (63%), and palpitations (32%) were only reported in PCC cohort. Comorbidities such as diabetes, dyslipidemia, and arterial hypertension were observed in all groups, but there were no significant differences between cohorts.

Immunomodulators represented the most common treatment used during acute SARS-CoV-2 infection for Severe (75%) and Critical (80%) participants, while the most common treatment for PCC participants was antibiotics (63%). Oxygen therapy was required in individuals from Severe (25%), Critical (60%), and PCC (11%) cohorts (p<0.001) during acute infection, while invasive mechanical ventilation was only required in individuals with Critical COVID-19 admitted to the ICU (75%). 20% of participants from the Critical cohort were exitus due to complications related to acute COVID-19 (ID numbers 43, 45, 47, and 51).

### Blood samples

Blood samples were collected at different time points depending on the cohort to evaluate the immune parameters during acute infection in participants with Severe and Critical COVID in comparison with participants with Mild COVID-19 who were already recovered, and participants diagnosed with PCC. Therefore, for participants with Severe and Critical COVID-19, blood samples were taken during the acute infection, when they were hospitalized and showed signs and symptoms characteristic of these conditions. Hence, the days from clinical onset to sample were 13 (IQR 9-17) and 25 days (IQR 12-35), respectively. Participants with Mild COVID-19 were recruited 85 days (IQR 80-95) after diagnosis of COVID-19, once there were completely recovered and showed no signs or symptoms of the disease. Therefore, they were controls for the return to normality of the immune response after acute infection, as it is expected that complete recovery occurs within 4-12 weeks after infection (16, 45–48). Due to individuals with Severe COVID-19 or underlying medical issues may retain some changes in the immune response beyond 6 months after infection (49), we recruited the participants with PCC 330 days (IQR 342-352) after COVID-19 diagnosis to avoid confusion with long-term sequelae of severe COVID-19 or Post-acute sequelae of COVID-19 (PASC) that may persist at least 6 months after the acute infection (50, 51) (**Table 1**). All PCC participants had mild COVID-19 presentation at the moment of diagnosis but since then they showed persistent signs and symptoms characteristic of PCC that did not substantially change over time.

### Lower levels of CD4+ Th1 cells with higher capacity to express IFN γ in participants with PCC

**Figure 1:**
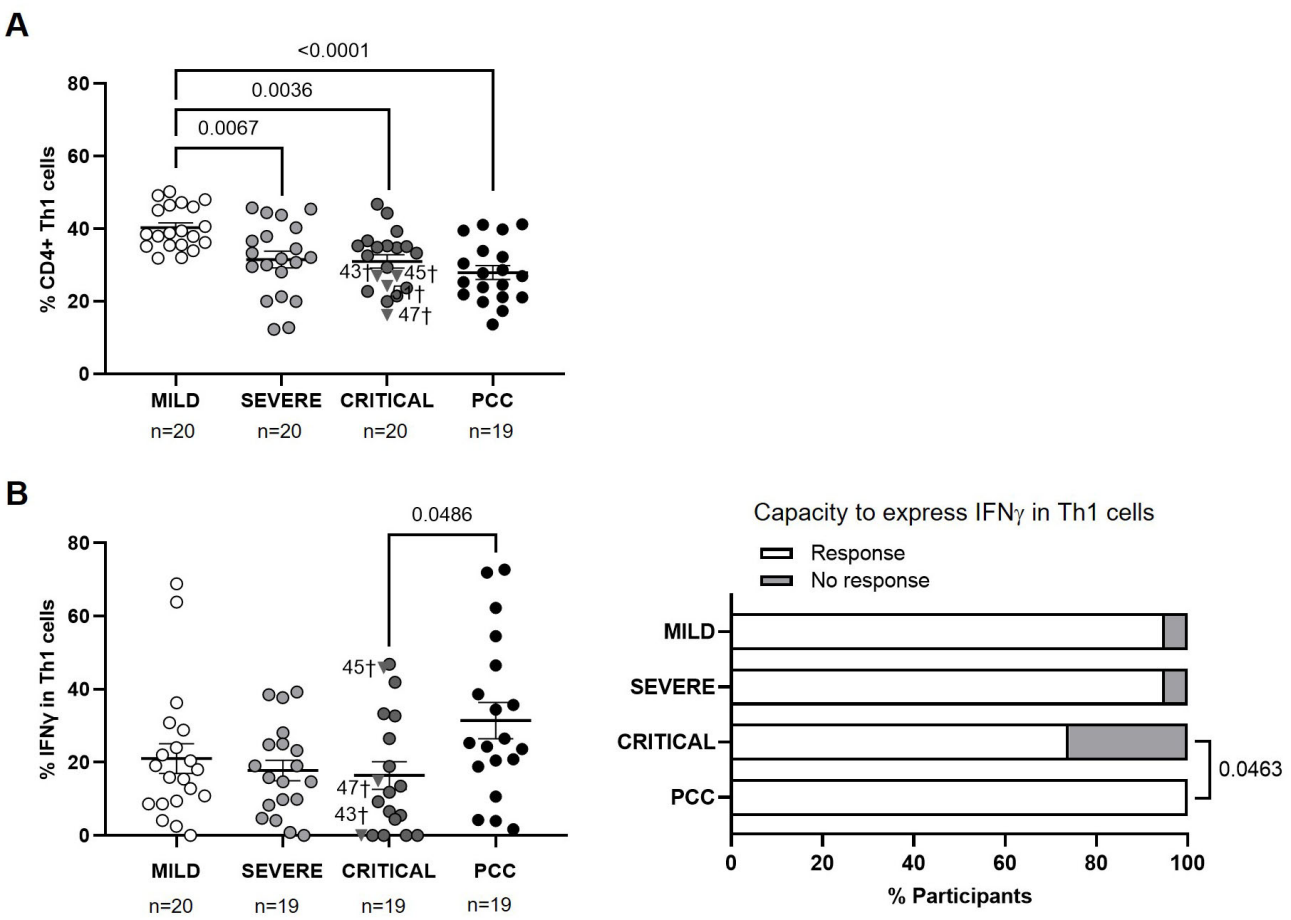
Levels of CD4+ Th1 cells and expression of IFNγ in individuals with acute COVID-19 and PCC. **(A)** Blood levels of CD4+ Th1 cells in individuals from each cohort of acute COVID-19 and PCC. **(B)** Intracellular expression of IFNγ in CD4+ Th1 cells upon stimulation in individuals from each cohort (left graph) and percentage of individuals whose CD4+ Th1 cells expressed (Response, open bar) or not (No response, light gray bar) IFNγ upon stimulation (right graph). Each dot in scatter plots corresponds to one sample and lines represent the mean ± standard error of the mean (SEM). Each symbol represents a different cohort: Mild COVID-19 (open circles), Severe COVID-19 (light gray circles), Critical COVID-19 (dark gray circles), and PCC (closed circles). Individuals from Critical cohort with exitus are identified with † symbol and their ID number: 43, 45, 47, and 51. Ordinary one-way ANOVA and Tukey post-test were applied to calculate the statistical significance between cohorts in scatter plots. Fisher´s exact test was used to calculate significance between cohorts in horizontal bar graphs. Significant p-values below 0.05 are represented.

Participants from Severe, Critical, and PCC cohorts showed lower levels of CD4+ Th1 cells than participants from Mild cohort (-1.3-fold, p=0.0067; -1.3-fold, p=0.0036; and -1.4-fold, p<0.0001, respectively) (**Figure 1A**). Participants from Critical cohort with exitus (ID numbers 43, 45, 47, and 51) showed Th1 levels below the average in their group (31.00 ± 8.20%).

CD4+ Th1 cells from participants with PCC showed 1.9-fold (p=0.0486) higher capacity to express IFNγ in response to activating stimuli than participants from Critical cohort, while no significant differences were found between the other cohorts (**Figure 1B**, left graph). 26% of participants from Critical cohort and 5% of participants from Mild and Severe cohorts were not able to express IFNγ in Th1 cells, while Th1 cells from 100% of participants with PCC were able to express IFNγ (p=0.0463) (**Figure 1B**, right graph).

The calculation of Pearson’s correlation between the levels of CD4+ Th1 and the expression of IFNγ in these cells from participants of different cohorts showed that there was a significant positive correlation in individuals from Mild (r=0.4737; p=0.0405) and PCC (r=0.5421; p=0.0165) cohorts (**Supplementary Figure 3**). In participants from Critical cohort, there was a significant negative correlation between the levels of CD4+ Th1 and the intracellular expression of IFNγ (r=-0.5245; p=0.0211).

### Lower levels of CD4+ Th2 cells with higher capacity to express IL-4 and IL-13 in participants with PCC

**Figure 2:**
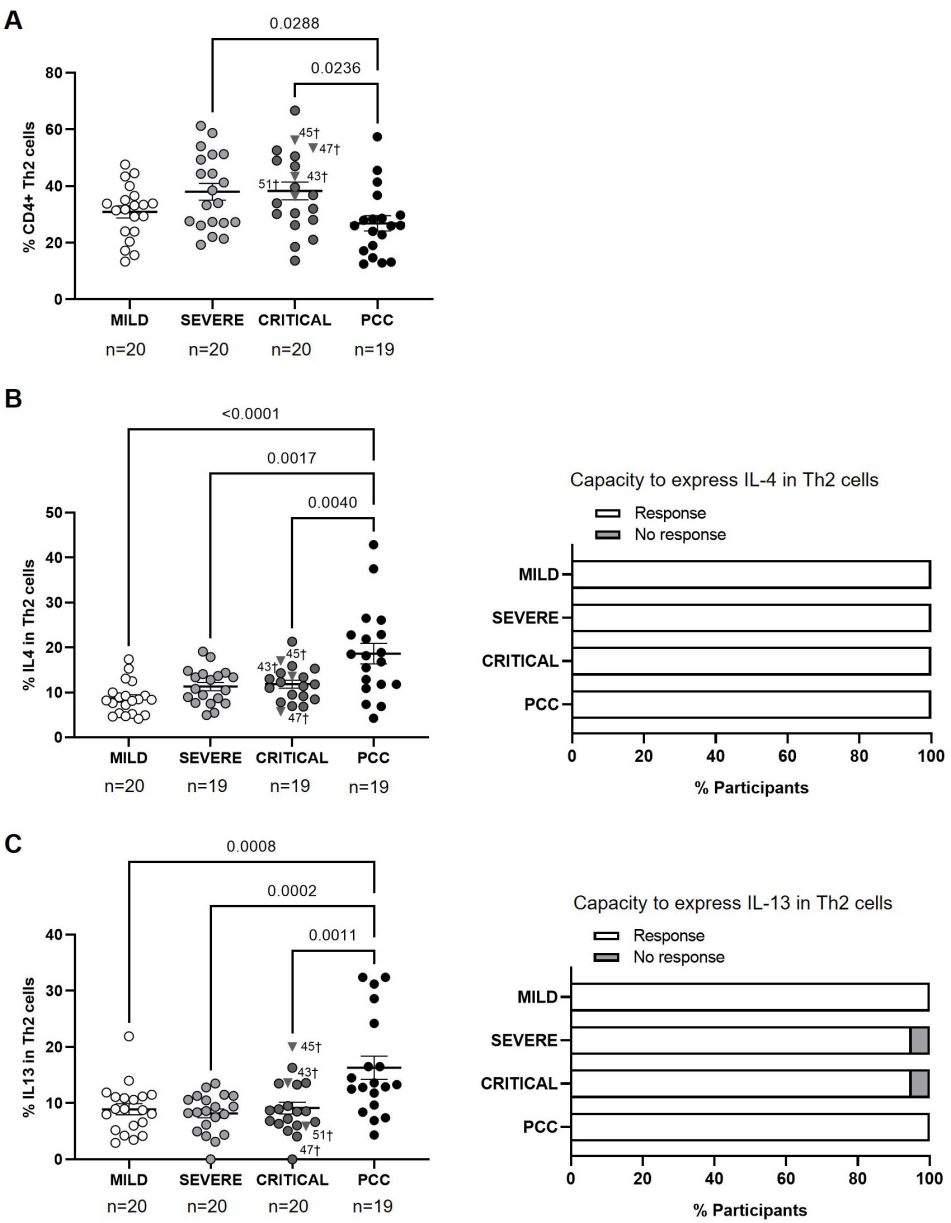
Levels of CD4+ Th2 cells and expression of IL-4 and IL-13 in individuals with acute COVID-19 and PCC. **(A)** Blood levels of CD4+ Th2 cells in individuals from each cohort of acute COVID-19 and PCC. Intracellular expression of IL-4 **(B)** and IL-13 **(C)** in CD4+ Th2 cells upon stimulation in individuals from each cohort (left graphs) and percentage of individuals whose CD4+ Th2 cells expressed (Response, open bar) or not (No response, light gray bar) these cytokines upon stimulation (right graph). Each dot in scatter plots corresponds to one sample and lines represent the mean ± SEM. Each symbol represents a different cohort: Mild COVID-19 (open circles), Severe COVID-19 (light gray circles), Critical COVID-19 (dark gray circles), and PCC (closed circles). Individuals from Critical cohort with exitus are identified with † symbol and their ID number: 43, 45, 47, and 51. Ordinary one-way ANOVA and Tukey post-test were applied to calculate the statistical significance between cohorts in scatter plots. Fisher´s exact test was used to calculate significance between cohorts in horizontal bar graphs. Significant p-values below 0.05 are represented.

Participants from Mild, Severe, and Critical cohorts showed similar levels of CD4+ Th2 cells, while participants with PCC showed significantly lower levels of these cells than individuals from Severe and Critical cohorts (-1.4-fold, p=0.0288; and -1.4-fold, p=0.0236, respectively) (**Figure 2A**). Three participants from Critical cohort with exitus (IDs 43, 45, and 47) showed Th2 levels above the average in their group (38.30 ± 13.88%).

The average expression of IL-4 and IL-13 in CD4+ Th2 cells after stimulation was higher in participants with PCC than in the other cohorts. The expression of IL-4 was 2.2- (p<0.0001), 1.7- (p=0.0017), and 1.6-fold (p=0.0040) higher in PCC participants in comparison with Mild, Severe, and Critical participants, respectively (**Figure 2B**, left graph), while the expression of IL-13 was 1.8- (p=0.0008), 2.0- (p=0.0002), and 1.8-fold (p=0.0011) higher in PCC participants (**Figure 2C**, left graph). CD4+ Th2 cells from all participants were able to express IL-4 in response to stimulation (**Figure 2B**, right graph), while only two individuals from the Severe and Critical cohorts were non-responders to express IL-13 (**Figure 2C**, right graph).

The calculation of Pearson’s correlation between the levels of CD4+ Th2 and the expression of IL-4 in these cells from the participants of the different cohorts showed that there was a significant, negative correlation in individuals with Mild (r=-0.4876; p=0.0401) and PCC (r=-0.5375; p=0.0261) (**Supplementary Figure 4**). There was no linear relationship between both variables in participants from Severe and Critical cohorts. We found no correlation between Th2 cells and the expression of IL-13 in none of the cohorts (**Supplementary Figure 5**).

### Absence of correlation between CD4+ Th1 and Th2 cell levels in participants with PCC

**Figure 3:**
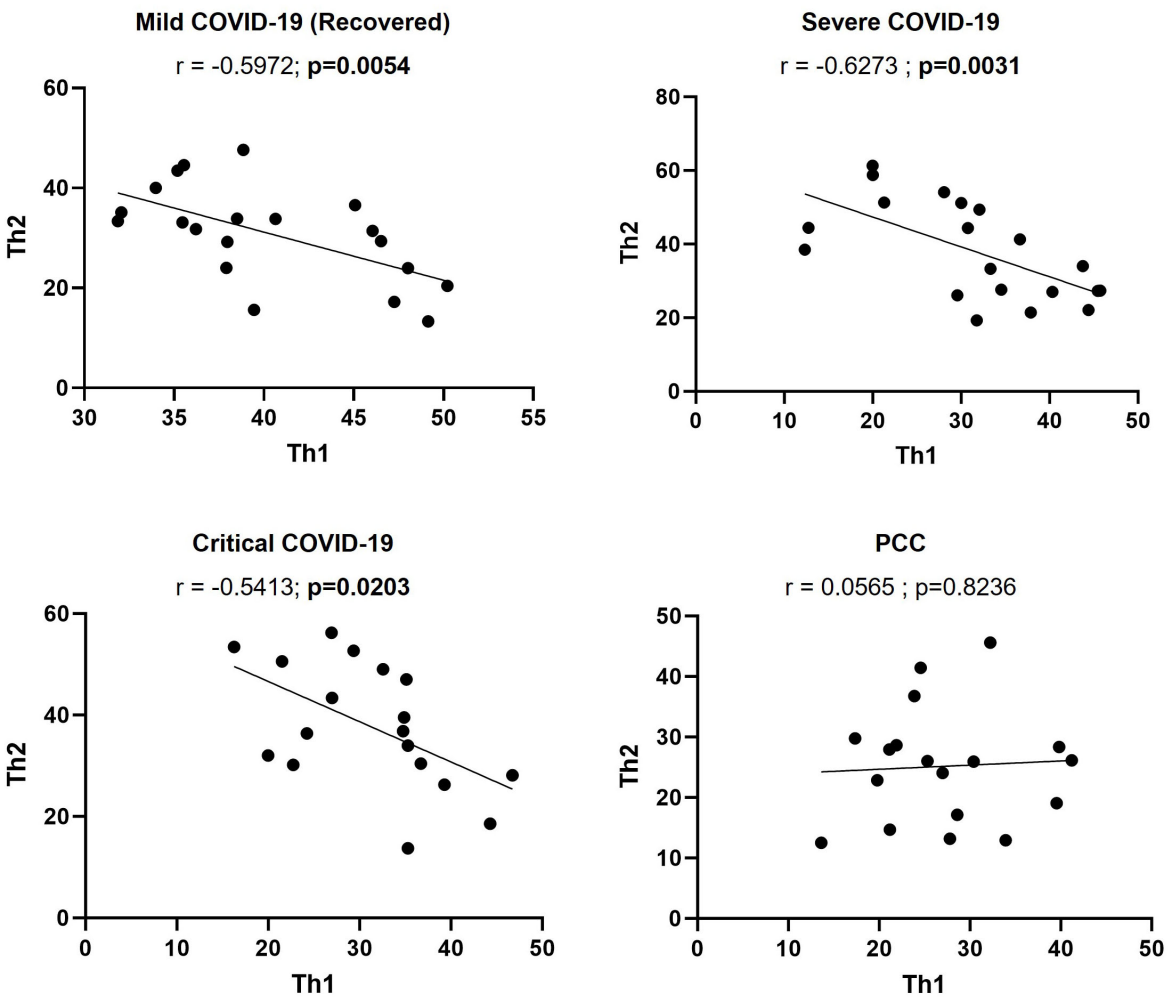
Correlation between the levels of CD4+ Th1 and Th2 cells in individuals with acute COVID-19 and PCC. Pearson’s coefficient r and p-values between the percentage of expression of CD4+ Th1 and Th2 cells were calculated for each cohort. Each dot corresponds to one sample and lines represent the linear regression.

The calculation of Pearson’s correlation between the levels of CD4+ Th1 and Th2 cells from participants of the different cohorts showed that there was a significant, negative correlation in individuals from Mild (r=-0.5972; p=0.0054), Severe (r=-0.6273; p=0.0031), and Critical cohorts (r=-0.5413; p=0.0203) (**Figure 3**), as expected (52). In participants with PCC, there was no linear association between the levels of CD4+ Th1 and Th2.

### Higher levels of CD4+ Th17 cells with reduced capacity to express IL-17A and IL-22 in participants with PCC

**Figure 4:**
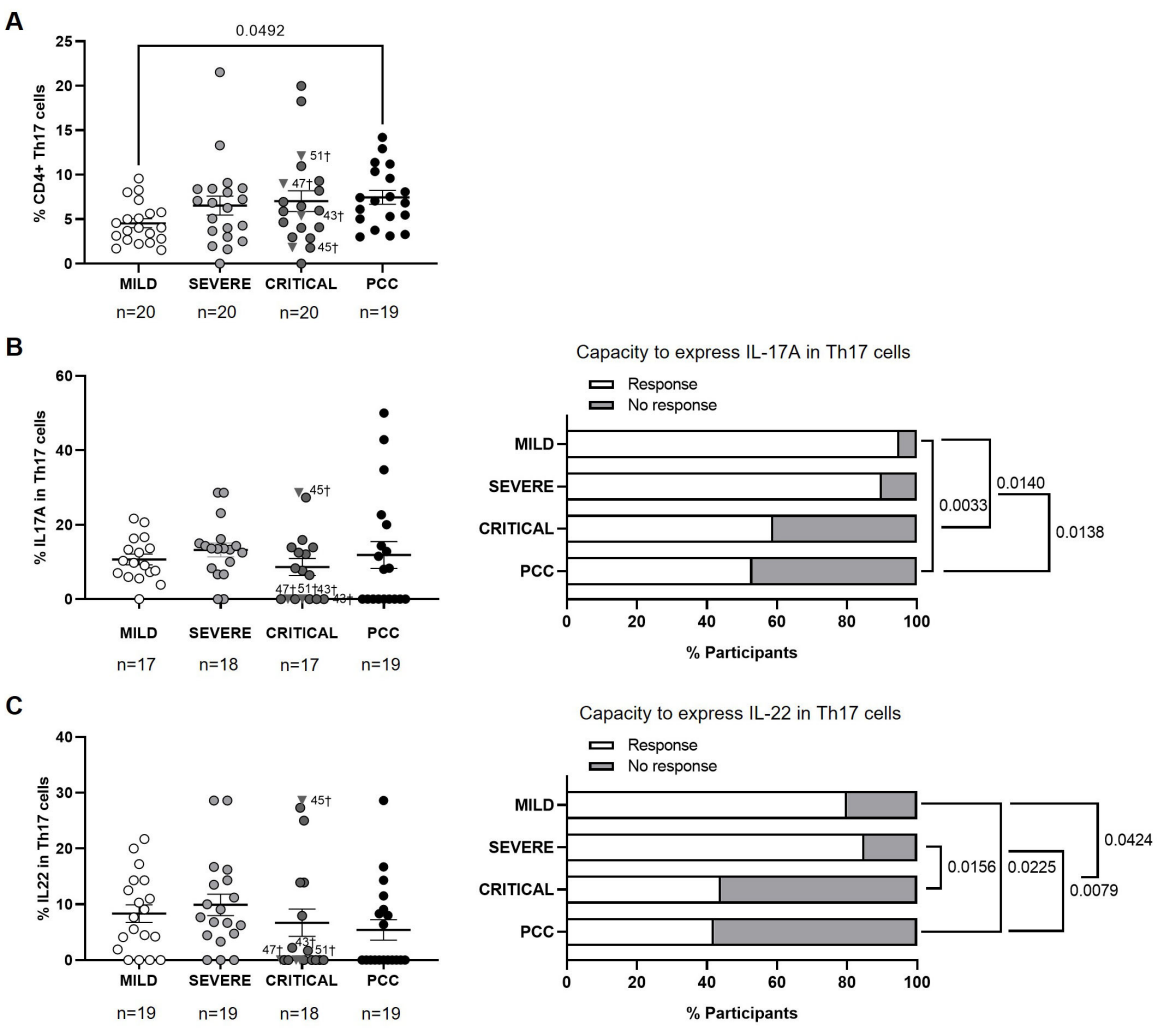
Levels of CD4+ Th17 cells and expression of IL-17A and IL-22 in individuals with acute COVID-19 and PCC. **(A)** Blood levels of CD4+ Th17 cells in individuals from each cohort of acute COVID-19 and PCC. Intracellular expression of IL-17A **(B)** and IL-22 **(C)** in CD4+ Th17 cells upon stimulation in individuals from each cohort (left graphs) and percentage of individuals whose CD4+ Th17 cells expressed (Response, open bar) or not (No response, light gray bar) these cytokines upon stimulation (right graph). Each dot in scatter plots corresponds to one sample and lines represent the mean ± SEM. Each symbol represents a different cohort: Mild COVID-19 (open circles), Severe COVID-19 (light gray circles), Critical COVID-19 (dark gray circles), and PCC (closed circles). Individuals from Critical cohort with exitus are identified with † symbol and their ID number: 43, 45, 47, and 51. Kruskal-Wallis test and Dunn’s multiple comparisons test were applied to calculate the statistical significance between cohorts in scatter plots. Fisher´s exact test was used to calculate significance between cohorts in horizontal bar graphs. Significant p-values below 0.05 are represented.

Participants from Mild, Severe, and Critical cohorts showed similar levels of CD4+ Th17 cells, while participants with PCC showed higher levels than Mild cohort (1.6-fold, p=0.0492) (**Figure 4A**).

No significant changes were observed between cohorts in the average capacity to express IL-17A or IL-22 in CD4+ Th17 cells (**Figures 4B, C**, left graphs). However, 47% of participants with PCC and 41% of participants from the Critical cohort showed CD4+ Th17 cells without capacity to express IL-17A in response to stimulation, in comparison with 10% and 5% of participants from the Severe and Mild cohorts, respectively (**Figure 4B**, right graph). The comparison between groups achieved significance in the comparison between PCC and Severe cohorts (p=0.0138), PCC and Mild cohorts (p=0.0033), and Mild and Critical cohorts (p=0.0140).

Regarding the expression of IL-22, 58% of participants from PCC cohort and 56% of participants from Critical cohort showed CD4+ Th17 cells without capacity to express IL-22 in response to stimulation, versus 15% and 20% of participants from Severe and Mild cohorts, respectively (**Figure 4B**, right graph). The comparison between cohorts achieved significance between PCC and Severe cohorts (p=0.0079), PCC and Mild cohorts (p=0.0225), Severe and Critical cohorts (p=0.0156), and Mild and Critical cohorts (p=0.0424). Three participants from Critical cohort with exitus (IDs 43, 47, and 51) did not express IL-17A or IL-22 in CD4+ Th9 cells in response to stimulation, while participant 45 with exitus from Critical cohort showed the highest expression of both cytokines.

### Higher levels of CD4+ Th9 cells with regular capacity to express IL-9 in participants with PCC

**Figure 5:**
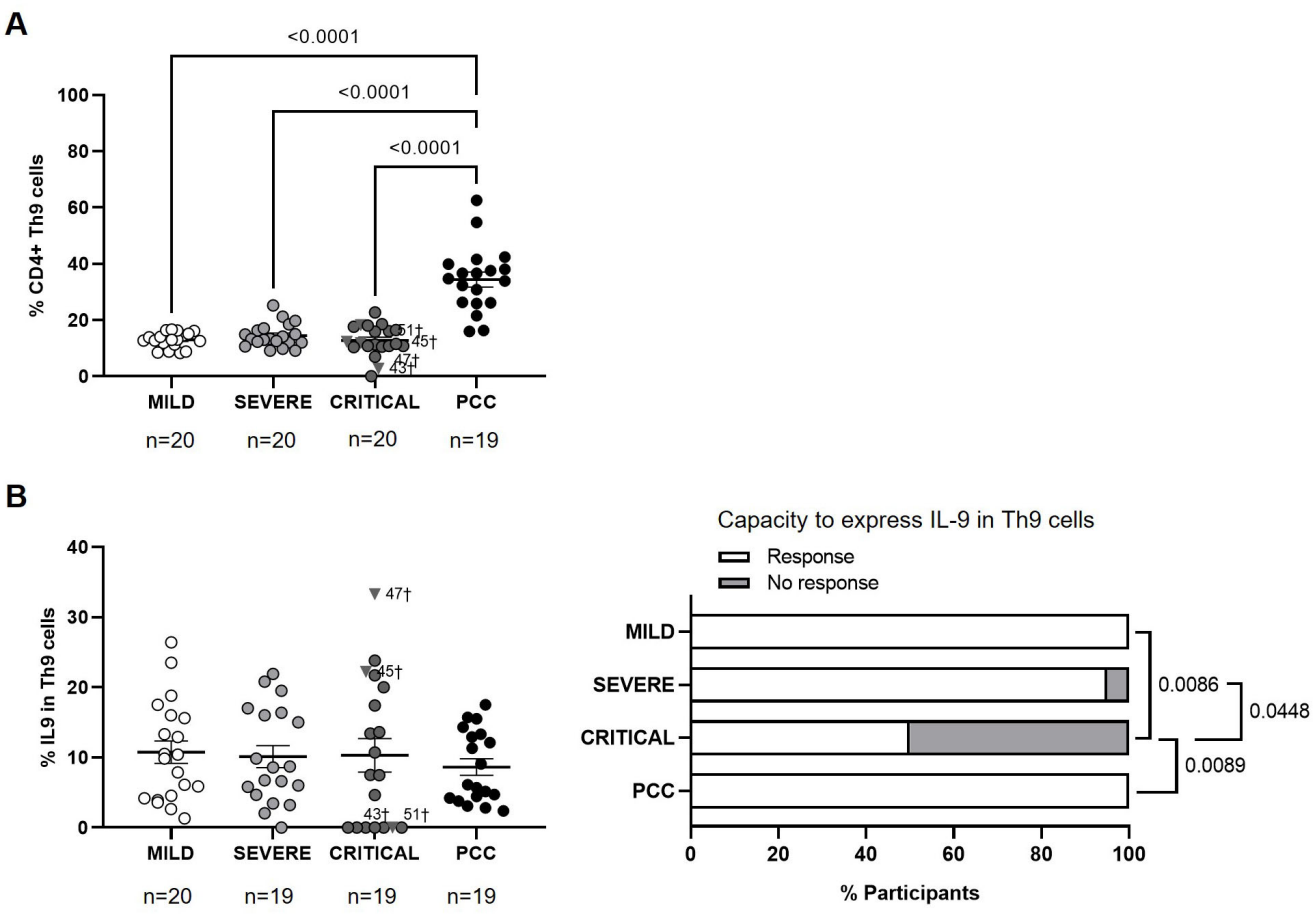
Levels of CD4+ Th9 cells and expression of IL-9 in individuals with acute COVID-19 and PCC. **(A)** Blood levels of CD4+ Th9 cells in individuals from each cohort of acute COVID-19 and PCC. **(B)** Intracellular expression of IL-9 in CD4+ Th9 cells upon stimulation in individuals from each cohort (left graphs) and percentage of individuals whose CD4+ Th9 cells expressed (Response, open bar) or not (No response, light gray bar) these cytokines upon stimulation (right graph). Each dot in scatter plots corresponds to one sample and lines represent the mean ± SEM. Each symbol represents a different cohort: Mild COVID-19 (open circles), Severe COVID-19 (light gray circles), Critical COVID-19 (dark gray circles), and PCC (closed circles). Individuals from Critical cohort with exitus are identified with † symbol and their ID number: 43, 45, 47, and 51. Ordinary one-way ANOVA and Tukey post-test were applied to calculate the statistical significance between cohorts in scatter plots. Fisher´s exact test was used to calculate significance between cohorts in horizontal bar graphs. Significant p-values below 0.05 are represented.

Participants from PCC cohort showed higher levels of CD4+ Th9 cells in comparison with participants from Mild, Severe, and Critical cohorts (2.7-, 2.4-, and 2.7-fold, respectively; p<0.0001) (**Figure 5A**). There were no significant changes in the average expression of IL-9 in CD4+ Th9 cells upon stimulation (**Figure 5B**, left graph), but 50% of participants from Critical cohort did not express IL-9 in response to stimuli and this difference was significant in the comparison with participants from PCC (p=0.0089), Severe (p=0.0448), and Mild (p=0.0086) cohorts (**Figure 5B**, right graph).

### Reduced capacity to express IL-13 and IL-22 from CD4+ Th22 cells of PCC

**Figure 6:**
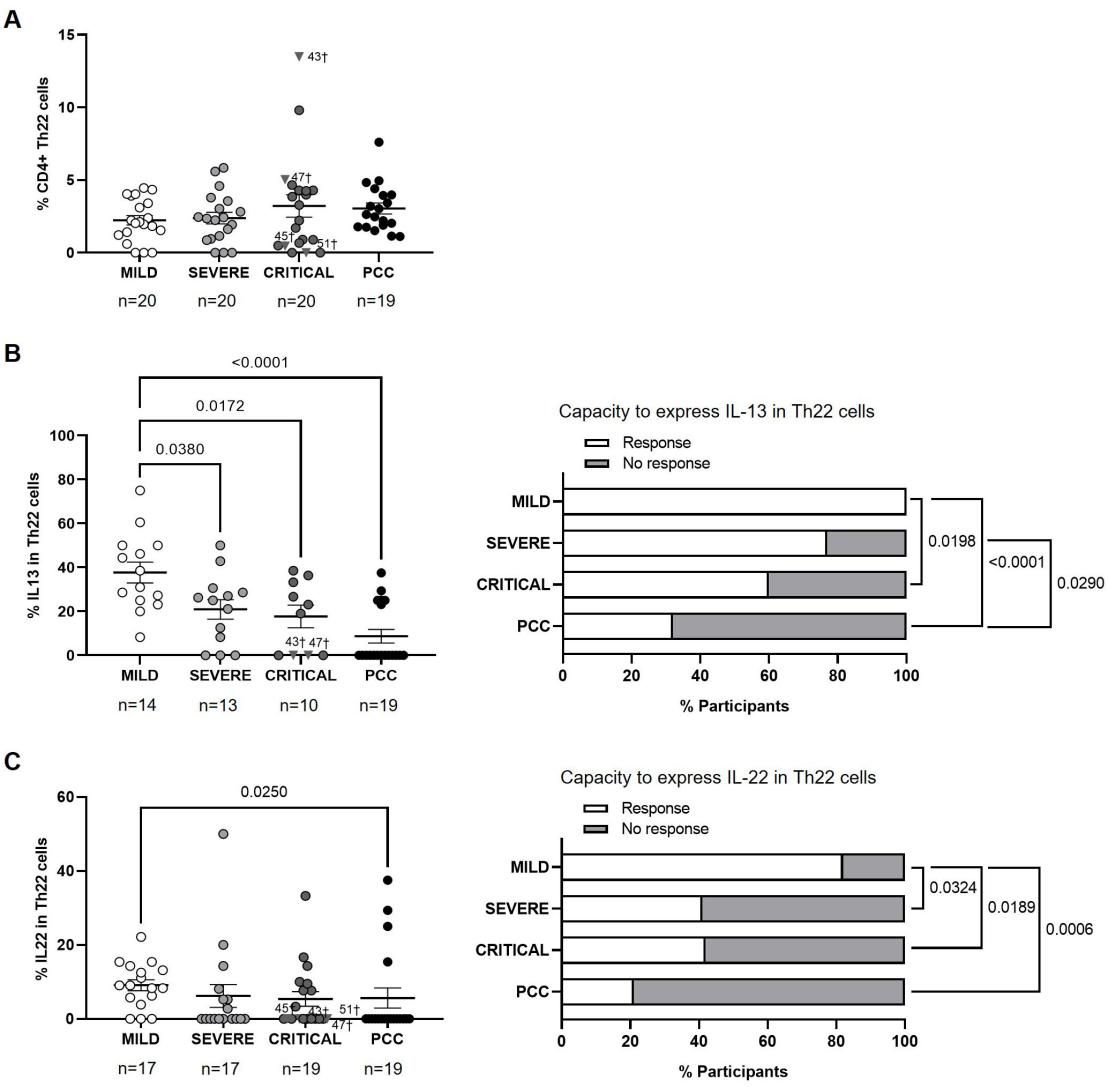
Levels of CD4+ Th22 cells and expression of IL-13 and IL-22 in individuals with acute COVID-19 and PCC. **(A)** Blood levels of CD4+ Th22 cells in individuals from each cohort of acute COVID-19 and PCC. Intracellular expression of 13 **(B)** and IL-22 **(C)** in CD4+ Th22 cells upon stimulation in individuals from each cohort (left graphs) and percentage of individuals whose CD4+ Th22 cells expressed (Response, open bar) or not (No response, light gray bar) these cytokines upon stimulation (right graph). Each dot in scatter plots corresponds to one sample and lines represent the mean ± SEM. Each symbol represents a different cohort: Mild COVID-19 (open circles), Severe COVID-19 (light gray circles), Critical COVID-19 (dark gray circles), and PCC (closed circles). Individuals from Critical cohort with exitus are identified with † symbol and their ID number: 43, 45, 47, and 51. Ordinary one-way ANOVA and Tukey post-test and Kruskal-Wallis test and Dunn’s multiple comparisons test were applied according to data normality to calculate the statistical significance between cohorts in scatter plots. Fisher´s exact test was used to calculate significance between cohorts in horizontal bar graphs. Significant p-values below 0.05 are represented.

No significant changes were observed in the levels of CD4+ Th22 cells between cohorts (**Figure 6A**). The average expression of IL-13 in CD4+ Th22 cells upon stimulation was 1.8- (p=0.0380), 2.1- (p=0.0172), and 4.3-fold (p<0.0001) lower in participants from Severe, Critical, and PCC cohorts, respectively, in comparison with Mild cohort (**Figure 6B**, left graph). 68% of participants from PCC cohort, 40% of participants from Critical cohort, and 23% of participants from Severe cohort did not express IL-13 in Th22 in response to stimulation. This difference was significant in the comparison between participants from PCC and Mild cohorts (p<0.0001), PCC and Severe cohorts (p=0.0290), and Mild and Critical cohorts (p=0.0198) (**Figure 6B**, right graph).

The average expression of IL-22 in CD4+ Th22 cells upon stimulation was 1.6-fold (p=0.0250) lower in participants from PCC cohort than Mild cohort (**Figure 6C**, left graph). CD4+ Th22 cells from 68% of participants from PCC cohort, 58% of participants from Critical cohort, 59% from Severe cohort, and 18% from Mild cohorts did not express IL-13 in response to stimuli (**Figure 6C**, right graph). There was significance in the comparisons between Mild cohort and Severe (p=0.0324), Critical (p=0.0189), and PCC cohorts (p=0.0006).

### Higher CD4+ Th cells polarization to proinflammatory responses in PCC cohort

The balance between the levels of all CD4+ Th cell populations was analyzed within each cohort by calculating the Pearson correlation coefficient r. There was a significant negative Th1/Th2 correlation in participants from Mild (r=-0.6000; p=0.0054) and Severe (r=-0.627; p=0.0030) cohorts (**Supplementary Figure 6**). Severe and PCC cohorts showed significant negative Th1/Th9 correlation (r=-0.4590; p=0.0420 and r=-0.7400; p=0.0002, respectively), while negative Th2/Th9 correlation was significant in Mild (r=-0.4200; p=0.0640) and Critical (r=-0.5000; p=0.0250) cohorts. Negative Th1/Th17 correlation was significant in Critical cohort (r=-0.4590; p=0.0420), while Th2/Th17 correlation was negative in Severe cohort (r=-0.5140; p=0.0200) and positive in PCC cohort (r=0.5600; p=0.0130). Finally, negative Th1/Th22 correlation was significant in Mild cohort (r=-0.4500; p=0.0460), while negative Th2/Th22 correlation was significant in Severe (r=-0.5000; p=0.0250) and Critical (r=-0.5230; p=0.0180) cohorts. Negative Th9/Th22 correlation was only significant in PCC cohort (r=-0.4600; p=0.0460).

### Role of CD4+ Th subsets and expression of related cytokines in different presentations of COVID-19

The role of blood levels of CD4+ Th cell subsets and their capacity to express related cytokines in the development of PCC and severe and critical forms of COVID-19 was analyzed by simple linear regression analysis and subsequent binary logistic regression analyses (OR). Simple linear regression analyses showed that CD4+ Th1, Th2, Th9, and Th17 levels, as well as the expression levels of IL-4 from CD4+ Th2 cells and IL-13 from CD4+ Th2 and Th22 cells presented a trend towards an association with the development and/or persistence of PCC, in comparison with Mild cohort (**Table 2A**). These results were confirmed by binary logistic regression analysis, suggesting that the levels of Th1 (OR 0.7855; 95% CI 0.6762 to 0.9122; p=0.0020), Th2 (OR 0.9319; 95% CI 0.8691 to 0.9992; p=0.0480), Th9 (OR 1.2996; 95% CI 1.0914 to 1.5476; p=0.0030), and Th17 (OR 1.4487; 95% CI 1.0941 to 1.9183; p=0.0100), as well as the expression levels of IL-4 from CD4+ Th2 cells (OR 1.2992; 95% CI 1.0943 to 1.5423; p=0.0030) and IL-13 from CD4+ Th2 (OR 1.2119; 95% CI 1.0346 to 1.4195; p=0.0170) and Th22 cells (OR 0.8905; 95% CI 0.8250 to 0.9612; p=0.0030) were positively correlated with the occurrence of PCC.

**Table 2:** Association between the levels of CD4+ Th cells and their capacity to express Th-related cytokines with the development of PCC (A), severe (B), or critical COVID-19 (C) was assessed in comparison with Mild COVID-19 using simple linear regression analysis and subsequent binary logistic regression analysis (OR).

A) PCC *versus* Mild COVID-19						
Variable	β	Simple linear 95% CI	p-value	OR	Binary logistic 95% CI	p-value
Th1	-12.3822	-17.0287 to -7.358	**<0.001**	0.7855	0.6762 to 0.9122	**0.002**
IFNγ in Th1	10.4020	-2.5682 to 23.3722	0.113	1.0277	0.9928 to 1.0638	0.120
Th2	-7.0620	-13.6989 to -0.4250	**0.038**	0.9319	0.8691 to 0.9992	**0.048**
IL-4 in Th2	10.0011	5.1938 to 14.8083	**<0.001**	1.2992	1.0943 to 1.5423	**0.003**
IL-13 in Th2	7.3831	2.8381 to 11.9281	**0.002**	1.2119	1.0346 to 1.4195	**0.017**
Th9	18.1041	12.4896 to 23.7186	**<0.001**	1.2996	1.0914 to 1.5476	**0.003**
IL-9 in Th9	-2.1078	-6.1620 to 1.9463	0.299	0.9445	0.8493 to 1.0500	0.293
Th17	2.9177	1.0550 to 4.7805	**0.003**	1.4487	1.0941 to 1.9183	**0.010**
IL-17A in Th17	1.1871	-7.0271 to 9.4013	0.771	1.0086	0.9535 to 1.0669	0.763
IL-22 in Th17	-2.9200	-7.8282 to 1.9882	0.235	0.9459	0.8633 to 1.0365	0.234
Th22	0.4148	-0.5045 to 1.3343	0.366	1.2417	0.7832 to 1.9687	0.357
IL-13 in Th22	-29.0250	-40.0969 to -17.9532	**<0.001**	0.8905	0.8250 to 0.9612	**0.003**
IL-22 in Th22	-3.4579	-9.9714 to 3.0556	0.288	0.9609	0.8931 to 1.0339	0.287
B) Severe *versus* Mild COVID-19						
Variable	β	Simple linear 95% CI	p-value	OR	Binary logistic 95% CI	p-value
Th1	-8.7655	-14.1038 to -3.4271	**0.002**	0.8690	0.7823 to 0.9652	**0.009**
IFNγ in Th1	-3.2501	-13.3699 to 6.8697	0.519	0.9858	0.9447 to 1.0286	0.511
Th2	7.1100	-0.2656 to 14.4856	0.058	1.0570	0.9962 to 1.1216	0.067
IL-4 in Th2	2.6742	0.2117 to 5.1368	**0.035**	1.2099	1.0059 to 1.4554	**0.043**
IL-13 in Th2	-0.7330	-3.2717 to 1.8057	0.562	0.9518	0.8086 to 1.1204	0.553
Th9	0.1820	-2.2258 to 2.5898	0.879	1.0136	0.8557 to 1.2006	0.875
IL-9 in Th9	-0.6194	-5.1565 to 3.9176	0.784	0.9867	0.8996 to 1.0822	0.777
Th17	0.9255	-0.9442 to 2.7952	0.323	1.1209	0.8966 to 1.4013	0.316
IL-17A in Th17	2.5648	-2.2426 to 7.3724	0.286	1.0576	0.9551 to 1.1711	0.281
IL-22 in Th17	1.5668	-3.4844 to 6.6181	0.533	1.0284	0.9435 to 1.1210	0.523
Th22	0.0315	-1.0137 to 1.0767	0.952	1.0125	0.6858 to 1.4946	0.950
IL-13 in Th22	-16.7608	-30.2453 to -3.2762	**0.017**	0.9389	0.8860 to 0.9950	**0.033**
IL-22 in Th22	-2.8870	-9.8550 to 4.0808	0.405	0.9678	0.8957 to 1.0456	0.407
C) Critical *versus* Mild COVID-19						
Variable	β	Simple linear 95% CI	p-value	OR	Binary logistic 95% CI	p-value
Th1	-9.2975	-13.8376 to -4.7573	**<0.001**	0.8211	0.7184 to 0.9385	**0.004**
IFNγ in Th1	-4.5990	-15.8699 to 6.6718	0.414	0.9838	0.9469 to 1.0223	0.406
Th2	7.3960	-0.2251 to 15.0171	0.057	1.0562	0.9963 to 1.1197	0.066
IL-4 in Th2	3.1827	0.7171 to 5.6482	**0.013**	1.2572	1.0341 to 1.5285	**0.022**
IL-13 in Th2	0.2080	-2.6935 to 3.1095	0.885	1.0107	0.8783 to 1.1630	0.882
Th9	-0.3285	-3.1060 to 2.4490	0.812	0.9817	0.8476 to 1.1371	0.806
IL-9 in Th9	-0.4383	-6.1921 to 5.3153	0.878	0.9941	0.9243 to 1.0691	0.874
Th17	2.4970	-0.0734 to 5.0674	0.057	1.2023	0.9790 to 1.4764	0.079
IL-17A in Th17	-2.0447	-7.5199 to 3.4305	0.452	0.9652	0.8822 to 1.0561	0.441
IL-22 in Th17	-1.6396	-7.5138 to 4.2345	0.575	0.9778	0.9060 to 1.0552	0.564
Th22	0.3060	-1.0153 to 1.6273	0.642	1.0788	0.7898 to 1.4737	0.633
IL-13 in Th22	-20.0092	-34.7782 to -5.2403	**0.010**	0.9276	0.8674 to 0.9920	**0.028**
IL-22 in Th22	-3.7089	-8.8679 to 1.4500	0.153	0.9331	0.8469 to 1.0281	0.162

CI, Confidence Interval; OR, Odds Ratio. Significant p-values are indicated in bold letters.

Same analyses were performed with data from Severe and Critical cohorts in comparison with Mild cohort (**Tables 2B, C**). Simple linear regression analyses showed that Th1 levels, and the expression levels of IL-4 from CD4+ Th2 cells and IL-13 from CD4+ Th22 cells presented a trend towards an association with the development of severe and critical forms of acute COVID-19, in comparison with individuals who fully recovered from mild COVID-19. These results were confirmed by binary logistic regression analysis, suggesting that the levels of Th1 (OR 0.8690; 95% CI 0.7823 to 0.9652; p=0.0090), as well as the expression levels of IL-4 from CD4+ Th2 cells (OR 1.2099; 95% CI 1.0059 to 1.4554; p=0.0430) and IL-13 from CD4+ Th22 cells (OR 0.9389; 95% CI 0.8860 to 0.9950; p=0.0330) were positively correlated with the occurrence of severe COVID-19 (**Table 2B**). Similarly, the levels of Th1 (OR 0.8211; 95% CI 0.7184 to 0.9385; p=0.0040), as well as the expression levels of IL-4 from CD4+ Th2 cells (OR 1.2572; 95% CI 1.0341 to 1.5285; p=0.0220) and IL-13 from CD4+ Th22 cells (OR 0.9276; 95% CI 0.8674 to 0.9920; p=0.0280) were positively correlated with the occurrence of critical COVID-19 (**Table 2C**).

## Discussion

PCC is a multisystemic condition characterized by the persistence of a wide variety of syndromes, often severe, that follow COVID-19, such as cardiovascular and hematologic alterations, type 2 diabetes, myalgic encephalomyelitis/chronic fatigue syndrome (ME/CFS), and dysautonomia, especially postural orthostatic tachycardia syndrome (POTS) (11). The immune deregulation is also an important hallmark of COVID-19 that can be very varied including T-cell function impairment and exhaustion, low levels of effector cells, and reduced numbers of antigen presenting cells (32, 53, 54), but also increased levels of activated B cells, non-classical monocytes, cytotoxic cells, and pro-inflammatory cytokines (17, 32, 55). This exacerbated immune response may be responsible not only for general symptoms like fatigue, myalgia, arthralgia, and peripheral neuropathy, but also for hypersensitivity reactions like erythematous and urticarial rash, and pulmonary symptoms like cough, chest pain, pneumonia, post-COVID interstitial lung disease, and dyspnea (56). In our cohort, 53% of participants with PCC had persistent dyspnea one year after SARS-CoV-2 infection. This proportion was more similar to individuals with severe and critical presentations of acute COVID-19 than to people who had mild symptoms and completely recovered during the first 4 weeks post-infection, supporting the notion that long-term persistence of symptoms after acute infection may occur in some individuals. Although comorbidities such as diabetes, dyslipidemia, and arterial hypertension have been related to the development of more severe forms of acute COVID-19 (57), we found no significant differences related to these comorbidities in the tendency to develop PCC in our cohort. Therefore, other risk factors should be involved and some of them could be related to an impaired immune response.

CD4+ Th cell polarization has repeatedly demonstrated its importance during the development of viral diseases, serving as coordinator of either cellular or humoral immune responses (58–61). Each CD4+ Th cell subset induces the best immune response that should be developed to eliminate the pathogens. Accordingly, CD4+ Th1 cells are essential for an adequate antiviral response during SARS-CoV-2 acute infection (62–64), while COVID-19 severity has been associated with a predominant extracellular response mostly mediated by high levels of CD4+ Th2 cells (65) and cytokines such as IL-2 and IL-6 (66) that are initiators of the “cytokine storm” and create a pro-inflammatory environment with low efficacy for viral clearance (67). Within CD4+ T cell subtypes, individuals with critical COVID-19 also present high levels of CD4+ regulatory T cells (Tregs) that are implicated in the contraction of the inflammatory immune response developed during acute infection (20, 66). Tregs are also altered in people with PCC, pointing at the existence of an immune deregulation that cannot be controlled and might be involved in the post-acute persistence of symptoms (68, 69). Due to the central role of CD4+ T cells in the polarization of the immune response and the control of chronic viral infections (58), more analyses are needed to evaluate the importance of CD4+ Th cell subsets in the development of different forms of acute or persistent COVID-19.

In our study, participants who recovered from mild COVID-19 showed higher levels of Th1 cells than other groups. This is to be expected, as individuals who reported mild symptomatology during infection should be able to trigger a better antiviral response polarized towards Th1 cells than those who suffered worse clinical outcomes (70–72). Moreover, although infection has been already resolved in these participants, the presence of SARS-CoV-2 produces a profound deregulation of the immune system that may persist up to 8 months post-infection even in individuals with mild presentation of COVID-19 (20). Although some studies did not find differences in the percentage of Th1 cells according to COVID-19 severity (29, 66), in our cohort of people with PCC the levels of CD4+ Th1 cells were lower than in people who recovered from mild COVID-19 and more similar to those from people who presented severe and critical forms of acute COVID-19, in which Th1 subset is usually underrepresented (73, 74). The antiviral activity of Th1 cells, measured by the expression of IFNγ, did not show significant differences between groups with acute disease, but these cells showed the highest capacity to express IFNγ in response to stimulation in people with PCC, proving their functionality. This is in accordance with previous studies that correlate disease severity to immune exhaustion profiles in Th1 cells but not to the total number of cells within the subpopulation (75). Therefore, despite the low levels of Th1 cells in individuals with PCC, these cells showed high capacity to express IFNγ, likely contributing to a proinflammatory state.

The polarization of CD4+ Th0 cells to Th1 subset usually occurs in response to intracellular pathogens, thereby interfering with polarization to Th2 population that is more associated with a humoral extracellular response mediated by the stimulation of B cells (76). Consequently, there was a significant negative Th2/Th1 correlation in individuals with acute forms of COVID-19. However, likely due to levels of Th2 were lower in people with PCC in comparison with individuals with severe and critical forms of COVID-19, we found no significant Th2/Th1 correlation in this cohort. Nevertheless, Th2 cells from people with PCC presented the highest capacity to express IL-4 and IL-13 within our cohorts. The release of IL-4 and IL-13 from Th2 cells is considered an important factor for low ventilation and death associated with COVID-19 and these cytokines are targets for immunotherapy agents such as dupilumab (77). Moreover, IL-4 and IL-13 activate the same signal transduction pathways to induce the production of IgE by B cells, which would contribute to the proinflammatory environment (78, 79). Accordingly, although the levels of CD4+ Th1 cells appeared to be a protective factor against the development of both acute and persistent forms of COVID-19, the expression of anti-inflammatory cytokines such as IL-4 in Th2 cells was revealed as a risk factor associated to the development of severe and critical COVID-19. Moreover, IL-4 could be related to low levels of Th1 cells as it would polarize the immune response towards an extracellular immune response (80–82). Several reports describe controversial data about plasma levels of IL-4 and IL-13 in individuals with PCC (77, 83, 84), but this is the first report about the higher capacity of Th2 cells from people with PCC to express these anti-inflammatory cytokines.

The development of proinflammatory CD4+ Th9 and Th17 subsets has been described during hypersensitivity and inflammatory reactions (85–87). However, once the antigen that has triggered this polarization wanes, these cell populations return to normal levels (88). In PCC, the levels of Th9 and Th17 subsets were significantly higher in comparison with acute forms of COVID-19, in agreement with previous reports about the role of higher levels of Th9 and Th17 cells in the pathophysiology of PCC (89, 90). Moreover, Th1/Th9 correlation was negative in individuals with severe COVID-19 and PCC, pointing to the existence of a more proinflammatory than antiviral scenario. In addition, Th17 cells from more than 50% of individuals with PCC showed no capacity to express cytokines such as IL-17A and IL-22, similar to participants with critical COVID-19. Due to the production of IL-17A from Th17 cells is essential for clearing extracellular pathogens, the inappropriate functionality of this subset has been linked to development of acute respiratory distress syndrome (ARDS) during acute COVID-19 (91) and it may also be related to an impaired viral clearance during PCC. Th2/Th17 correlation was positive in people with PCC, which in the absence of a negative Th1/Th2 correlation could also contribute to the proinflammatory state. In fact, the levels of Th9 and Th17, as well as the expression of IL-4 and IL-13 from Th2 cells, which would counteract Th1 polarization, were appointed as risk factors in the development of PCC.

Finally, although there was no significant difference in the levels of CD4+ Th22 cell subset in people with PCC in comparison with participants with acute forms of COVID-19, the highest proportion of individuals in which Th22 cells showed no capacity to express IL-13 or IL-22 in response to stimuli were those in the PCC cohort. Th22 cell subset exhibits protective anti-inflammatory properties that can promote immunity against infection by HIV (92) and respiratory viruses such as influenza, respiratory syncytial virus (RSV), and SARS-CoV-2 (93, 94). The expression of IL-13 and IL-22 from Th22 subset appeared to be a protective factor in both acute and persistent COVID-19 (95). In fact, IL-13 may reduce the risk of SARS-CoV-2 infection in airway epithelium by decreasing the expression of its main receptor ACE-2 in ciliated cells and increasing the secretion of mucin and glycocalyx in the periciliary layer, which acts as a physical barrier against the virus attachment (65). The role of Th22 cells and IL-22 in the pathophysiology of PCC is not fully understood but IL-22-induced signaling pathway may switch from protective to pathogenic as COVID-19 progresses (95) due to although it acts on epithelial cells to promote tissue protection and regeneration, IL-22 may also elicit pro-inflammatory effects, contributing to disease pathology (96). The low capacity of Th22 cells from people with PCC to express IL-13 and IL-22 may increase tendency to reinfections, producing impaired epithelial regeneration that has been related to a higher susceptibility to develop PCC (97, 98). Significant correlations between Th22 cell subsets and other Th populations were very varied within our cohorts and although people who recovered from mild COVID-19 presented a significant negative Th1/Th22 correlation, people with severe and critical COVID-19 showed a significant negative Th2/Th22 correlation, while individuals with PCC showed a significant negative Th9/Th22 correlation.

One potential limitation of this study is that diagnostic tests were not performed at the moment of sampling to discard a possible asymptomatic infection with SARS-CoV-2.

In conclusion, people with PCC showed a skewed polarization of CD4+ Th subsets with altered functionality that was more similar to individuals with severe and critical presentations of acute COVID-19 than to people who fully recovered from mild disease. This profile presented lower polarization towards Th1-mediated antiviral immune response in the absence of significant Th2/Th1 negative correlation. In addition, higher levels of proinflammatory Th9 and Th17 cell subsets were observed in comparison with acute forms of COVID-19, with a reduced capacity to express anti-inflammatory cytokines related to endothelial regeneration such as IL-22. These results pointed to the possibility that individuals with PCC may present an impaired capacity to develop an adequate immune response against SARS-CoV-2 infection that may persist over time, causing a long-term pro-inflammatory environment.

## Data Availability

The original contributions presented in the study are included in the article/**Supplementary Material**. Further inquiries can be directed to the corresponding author.
