# Supplementary material for: Different polarization and functionality of CD4+ T helper subsets in people with post-COVID condition
Source: Front Immunol. 2024 Aug 27;15:1431411. doi: 10.3389/fimmu.2024.1431411 (PMC11385313; doi:10.3389/fimmu.2024.1431411)
Supplement: Supplementary file 1 [file Table1.docx]

**Supplementary Table 1.** Clinical characteristics of individuals with different presentations of COVID-19 who were recruited at the Primary Healthcare Center Dr Pedro Laín Entralgo (Madrid) (Non-hospitalized) and Hospital Universitario Ramon y Cajal (Madrid) (Hospitalized).

| **ID** | **Severity of COVID-19** | **Age**  **(years)** | **Gender**  **(M/F)** | **Exitus**  **(Y/N)** | **Signs and symptoms** | | | | | | | | | **Therapy for COVID-19** | | | **Co-morbidities** | | | **Hospitalization** | | **Days from clinical onset to sample** |
| --- | --- | --- | --- | --- | --- | --- | --- | --- | --- | --- | --- | --- | --- | --- | --- | --- | --- | --- | --- | --- | --- | --- |
|  |  |  |  |  | **Cough**  **(Y/N)** | **Dyspnea (Y/N)** | **Fever**  **(Y/N)** | **Pneumonia**  **(Y/N)** | **Lethargy**  **(Y/N)** | **Asthenia**  **(Y/N)** | **Palpitations**  **(Y/N)** | **Arrythmia**  **(Y/N)** | **DIC**  **(Y/N)** | **Treatment** | **Oxygen therapy** | **IMV**  **(Y/N)** | **HTA**  **(Y/N)** | **Dyslipidemia**  **(Y/N)** | **Diabetes mellitus**  **(Y/N)** | **LOS (days)** | **ICU**  **stay**  **(days)** |  |
| 1 | Mild | 28 | M | N | Y | N | Y | N | N | Y | N | N | N | - | N/A | N/A | N | N | N | N/A | N/A | 36 |
| 2 | Mild | 59 | M | N | N | N | Unk | N | N | N | N | N | N | - | N/A | N/A | N | N | N | N/A | N/A | 107 |
| 3 | Mild | 38 | M | N | N | N | Y | N | N | N | N | N | N | - | N/A | N/A | N | N | N | N/A | N/A | Unk |
| 4 | Mild | 70 | M | N | N | N | Unk | N | N | N | N | N | N | - | N/A | N/A | Y | N | N | N/A | N/A | Unk |
| 5 | Mild | 26 | M | N | N | N | Y | N | N | Y | N | N | N | - | N/A | N/A | N | N | N | N/A | N/A | 91 |
| 6 | Mild | 32 | M | N | Y | N | Y | Unk | N | N | N | N | N | - | N/A | N/A | N | N | N | N/A | N/A | 95 |
| 7 | Mild | 47 | M | N | N | N | Y | N | N | Y | N | N | N | - | N/A | N/A | N | N | N | N/A | N/A | 97 |
| 8 | Mild | 28 | M | N | Y | N | Y | N | N | Y | N | N | N | - | N/A | N/A | N | N | N | N/A | N/A | 79 |
| 9 | Mild | 28 | M | N | N | N | N | Unk | N | N | N | N | N | - | N/A | N/A | N | N | N | N/A | N/A | Unk |
| 10 | Mild | 64 | M | N | Y | Y | Unk | N | N | Y | N | N | N | HCQ | N/A | N/A | N | Y | Y | N/A | N/A | 75 |
| 11 | Mild | 35 | F | N | Y | N | N | N | N | Y | N | N | N | - | N/A | N/A | N | N | N | N/A | N/A | 89 |
| 12 | Mild | 59 | F | N | Y | Y | Y | N | N | Y | N | N | N | - | N/A | N/A | N | N | N | N/A | N/A | 90 |
| 13 | Mild | 25 | F | N | Y | N | Y | Unk | Y | Y | N | N | N | - | N/A | N/A | N | Y | N | N/A | N/A | 99 |
| 14 | Mild | 36 | F | N | N | N | N | N | N | Y | N | N | N | - | N/A | N/A | Y | N | N | N/A | N/A | 95 |
| 15 | Mild | 71 | F | N | Y | N | Y | N | N | Y | N | N | N | - | N/A | N/A | Y | Y | N | N/A | N/A | 95 |
| 16 | Mild | 54 | F | N | N | N | Y | N | N | N | N | N | N | - | N/A | N/A | N | N | N | N/A | N/A | 84 |
| 17 | Mild | 48 | F | N | Y | Y | Y | Y | N | Y | N | N | N | - | N/A | N/A | N | N | N | N/A | N/A | Unk |
| 18 | Mild | 52 | F | N | Y | N | Y | Unk | Unk | Y | N | N | N | - | N/A | N/A | N | N | N | N/A | N/A | 72 |
| 19 | Mild | 27 | F | N | Y | N | Unk | Unk | N | N | N | N | N | - | N/A | N/A | N | N | N | N/A | N/A | 81 |
| 20 | Mild | 63 | F | N | N | N | Unk | Unk | N | N | N | N | N | - | N/A | N/A | Y | N | N | N/A | N/A | Unk |
| 21 | Severe | 57 | M | N | N | N | Y | N | N | N | N | N | N | LMWH | N | N | N | N | N | 6 | N/A | 14 |
| 22 | Severe | 31 | M | N | N | N | Y | Y | N | N | N | N | N | DEX, TCZ, LMWH | N | N | N | N | N | 7 | N/A | 16 |
| 23 | Severe | 27 | M | N | N | N | Y | Y | N | N | N | N | N | CsA, MEM, Tec, AmB | N | N | N | N | N | 31 | N/A | 19 |
| 24 | Severe | 42 | M | N | Y | Y | Y | Y | Y | N | N | N | N | DEX, CTX | N | N | Y | N | N | 6 | N/A | 12 |
| 25 | Severe | 50 | M | N | Y | N | N | Y | N | N | N | N | N | HCQ | N | N | Y | N | N | 10 | N/A | 3 |
| 26 | Severe | 50 | M | N | Y | N | N | N | N | N | N | N | N | - | N | N | Y | Y | Y | 60 | N/A | 1 |
| 27 | Severe | 49 | M | N | N | Y | Y | Y | N | N | N | N | N | DEX, RDV | N | N | N | N | N | 6 | N/A | 12 |
| 28 | Severe | 62 | M | N | Y | Y | Y | Y | N | Y | N | N | N | LMWH, IPRA | N | N | N | N | N | 12 | N/A | 11 |
| 29 | Severe | 51 | M | N | Y | Y | Y | Y | N | N | N | N | N | DEX, LMWH | Nasal glasses | N | N | N | N | 4 | N/A | 13 |
| 30 | Severe | 58 | M | N | N | N | Y | Y | Y | N | N | N | N | TCZ, PBZ, DEX | Nasal glasses | N | Y | Y | N | 7 | N/A | 5 |
| 31 | Severe | 59 | F | N | Y | Y | Y | N | N | N | N | N | N | HCQ, AZM | Nasal glasses | N | N | Y | Y | 10 | N/A | 32 |
| 32 | Severe | 46 | F | N | Y | Y | N | Y | N | N | N | N | N | DEX | N | N | N | N | N | 4 | N/A | 10 |
| 33 | Severe | 32 | F | N | Y | N | Y | Y | N | N | N | N | N | DEX, AZM | N | N | N | N | N | 3 | N/A | 19 |
| 34 | Severe | 45 | F | N | N | Y | Y | Y | N | N | N | N | N | TCZ, DEX, Pred | Reservoir | N | N | Y | N | 9 | N/A | 7 |
| 35 | Severe | 46 | F | N | N | Y | Y | Y | N | N | N | N | N | DEX, CTX, LMWH | N | N | N | N | N | 6 | N/A | 7 |
| 36 | Severe | 19 | F | N | N | N | Y | Y | N | Y | N | N | N | - | N | N | N | N | N | 14 | N/A | 14 |
| 37 | Severe | 52 | F | N | Y | N | Y | Y | N | N | N | N | N | DEX, TCZ, LMWH | Nasal glasses | N | Y | N | Y | 19 | N/A | 21 |
| 38 | Severe | 54 | F | N | N | Y | Y | Y | N | N | N | N | N | DEX, ABT | N | N | N | Y | N | 11 | N/A | 20 |
| 39 | Severe | 53 | F | N | N | Y | N | Y | N | N | N | N | N | DEX, LMWH | N | N | N | N | N | 4 | N/A | 12 |
| 40 | Severe | 57 | F | N | N | Y | Y | Y | N | Y | N | N | N | DEX | N | N | Y | N | N | 5 | N/A | 13 |
| 41 | Critical | 36 | M | N | Y | Y | Y | Y | Y | Y | N | N | N | DEX, ABT, LMWH | N | Y | N | N | N | 30 | 13 | 12 |
| 42 | Critical | 36 | M | N | Y | Y | Y | Y | N | N | N | N | N | - | N | Y | N | N | N | 13 | 6 | 16 |
| 43 | Critical | 52 | M | Y | Y | Y | Y | Y | N | N | N | N | N | LPV/r, AZM | Reservoir | Y | Y | N | N | 48 | 49 | 34 |
| 44 | Critical | 59 | M | N | Y | Y | Y | Y | N | N | N | N | N | HCQ, LPV/r, AZM | Reservoir/ Nasal glasses | Y | Y | N | N | 45 | 7 | 44 |
| 45 | Critical | 48 | M | Y | Y | Y | N | N | N | Unk | N | N | N | MEM, Lzd, VOR, AMK, LMWH | N | Y | N | N | N | 17 | 18 | 22 |
| 46 | Critical | 46 | M | N | N | Y | Y | Y | N | N | N | N | N | - | N | Y | N | N | N | 17 | 14 | 10 |
| 47 | Critical | 54 | M | Y | N | Y | Y | Y | Y | N | N | N | N | DEX, TCZ, CTX, Pred | NIV | N | Y | Y | Y | 90 | 37 | 8 |
| 48 | Critical | 40 | M | N | Unk | Unk | Y | Y | N | N | N | N | N | HCQ, AZM | Nasal glasses | Y | U | U | U | 63 | 45 | 71 |
| 49 | Critical | 50 | M | N | Y | N | N | Y | Y | N | N | N | N | HCQ, LPV/r, AZM | Reservoir/ Nasal glasses | Y | Y | N | N | 52 | 13 | 48 |
| 50 | Critical | 52 | M | N | N | Y | Y | Y | N | N | N | N | N | HCQ, LPV/r, AZM | Nasal glasses | Y | Y | N | N | 42 | 39 | 50 |
| 51 | Critical | 47 | F | Y | Y | Y | N | Y | N | N | N | N | N | DEX, ABT | NIV | N | N | Y | Y | 44 | 8 | 25 |
| 52 | Critical | 54 | F | N | N | N | Y | Y | N | N | N | N | N | DEX, TCZ, ABT | N | Y | N | N | N | 85 | 60 | 19 |
| 53 | Critical | 58 | F | N | Y | Y | Y | Y | N | Y | N | N | N | HCQ, LPV/r, AZM | Reservoir/ Nasal glasses | Y | N | N | N | 96 | 42 | 38 |
| 54 | Critical | 56 | F | N | Y | N | N | Y | N | N | N | N | N | HCQ, LPV/r | Reservoir/ Nasal glasses | Y | N | N | N | 81 | 37 | 18 |
| 55 | Critical | 59 | F | N | Y | Y | Y | Y | N | N | N | N | N | TZP, Pred, LMWH | NIV | N | N | N | N | 24 | 9 | 14 |
| 56 | Critical | 63 | F | N | Y | N | Y | Y | N | Y | N | N | N | DEX, LMWH | N | Y | N | N | N | Unk | Unk | 12 |
| 57 | Critical | 60 | F | N | Y | N | N | Y | Y | Y | N | N | N | Pred, LMWH, CTX | NO | Y | N | N | N | 32 | 19 | 7 |
| 58 | Critical | 62 | F | N | N | Y | Y | Y | Y | Y | N | N | N | DEX, RDV, LMWH | Reservoir | N | Y | N | Y | 27 | Unk | 17 |
| 59 | Critical | 62 | F | N | Y | Y | Y | Y | Y | Y | N | N | N | DEX, ABT | NO | Y | Y | Y | Y | 80 | 57 | 17 |
| 60 | Critical | 61 | F | N | N | Y | Y | Y | N | N | N | N | N | Pred, ABT, LMWH | NIV | N | N | Y | N | 82 | 18 | 11 |
| 61 | PCC | 33 | F | N | N | >9 mo. | Y | Y | >9 mo. | >9 mo. | >9 mo. | >9 mo. | N | APAP, ABT, LMWH. LPV/r | N | N | N | N | N | N | N | 346 |
| 61 | PCC | 31 | F | N | N | >9 mo. | N | Y | >9 mo. | >9 mo. | N | N | N | ABT, CTX, DEX | N | N | N | N | N | N | N | 352 |
| 62 | PCC | 45 | F | N | N | 6-9 mo. | Y | Y | Unk | >9 mo. | N | 6-9 mo. | N | LEV, AVK, DEX | N | N | N | N | N | 3 | N | 347 |
| 63 | PCC | 48 | F | N | N | >9 mo. | Y | Unk | >9 mo. | >9 mo. | N | >9 mo. | N | RDV | N | N | N | Y | N | N | N | 358 |
| 64 | PCC | 55 | F | N | >9 mo. | N | N | N | >9 mo. | >9 mo. | 6-9 mo. | >9 mo. | N | Amitrip, IFN-β | N | N | N | N | N | N | >9 mo. | 348 |
| 65 | PCC | 50 | F | N | 6-9 mo. | N | N | N | Unk | 6-9 mo. | N | N | N | APAP | Nasal glasses | N | N | N | N | 6-9mo. | 6-9mo. | 192 |
| 66 | PCC | 33 | M | N | >9 mo. | N | Y | N | < 3 mo. | >9 mo. | N | < 3 mo. | N | LEV, DEX | N | N | N | N | N | N | N | Unk |
| 67 | PCC | 57 | F | N | >9 mo. | N | N | N | >9 mo. | >9 mo. | N | N | N | ABT, LMWH, FPV | N | N | N | N | N | >9 mo. | >9 mo. | 351 |
| 68 | PCC | Unk | F | N | >9 mo. | N | Y | N | >9 mo. | >9 mo. | >9 mo. | >9 mo. | N | MTM, LEV | N | N | N | N | N | N | N | 194 |
| 69 | PCC | 46 | F | N | >9 mo. | >9 mo. | N | N | >9 mo. | 3-6 mo. | N | 6-9 mo. | N | APAP, MTM, DKT, DAA, TFV, RDV | NIV | N | N | N | N | N | 3-6mo. | 345 |
| 70 | PCC | 42 | F | N | Unk | N | Y | N | >9 mo. | Unk | N | N | N | MTM, RDV | N | N | N | N | N | N | N | 341 |
| 71 | PCC | 46 | F | N | 3-6 mo. | 3-6 mo. | Y | N | >9 mo. | >9 mo. | N | 3-6 mo. | N | ABT | N | N | Y | Y | N | N | >9 mo. | 349 |
| 72 | PCC | 41 | F | N | >9 mo. | mo. | N | N | 6-9 mo. | >9 mo. | >9 mo. | N | N | APAP, IBU, DEX, Vit D | N | N | N | N | N | N | N | 347 |
| 73 | PCC | 41 | F | N | >9 mo. | 6-9 mo. | N | N | 6-9 mo. | >9 mo. | >9 mo. | N | N | APAP, IBU, DEX, Vit D | N | N | N | N | N | N | N | 347 |
| 74 | PCC | 37 | F | N | >9 mo. | N | Y | N | >9 mo. | >9 mo. | N | N | N | LMWH | N | N | N | N | N | N | N | 311 |
| 75 | PCC | 39 | F | N | >9 mo. | N | Y | N | >9 mo. | >9 mo. | >9 mo. | >9 mo. | Y | ABT, FPV | Nasal glasses/ NIV | N | N | N | N | >9 mo. | >9 mo. | 348 |
| 76 | PCC | 44 | F | N | >9 mo. | >9 mo. | Y | N | >9 mo. | >9 mo. | N | N | N | ABT | N | N | N | Y | N | N | N | 369 |
| 77 | PCC | Unk | F | N | <3 mo. | >9 mo. | Y | Y | >9 mo. | >9 mo. | N | <3 mo. | N | MTM, CTX, AZM, AVK, TFV, Vit D | N | N | N | N | N | N | N | 352 |
| 78 | PCC | 33 | F | N | >9 mo. | N | N | N | >9 mo. | >9 mo. | >9 mo. | >9 mo. | N | AZM, RXL, LPV/r | IMV | Y | N | N | N | >9 mo. | >9 mo. | 334 |
| 79 | PCC | 40 | F | N | <3 mo. | >9 mo. | Y | Y | >9 mo. | >9 mo. | N | <3 mo. | N | LEV, DEX | IMV | Y | Y | Y | Y | N | N | 360 |

ABT, Antibiotics; AmB, Anfotericin B; Amitrip, AMK, Amikacin; Amitriptyline; APAP, Acetaminophen; AVK, Antivitamin K; AZM, Azithromycin; CsA, Cyclosporine; CTX, Ceftriaxone; DAA, Direct Action Anticoagulants; DEX, Dexamethasone; DIC, Disseminated intravascular coagulation; DKT, Dexketoprofen; F, Female; FPV, Favipiravir; HCQ, Hydroxychloroquine; HTA, Hypertension; IBU, Ibuprophen; ICU, Intensive care unit; IMV, Invasive Mechanical Ventilation; IPRA, Ipratropium Bromide; LEV, Levofloxacin; LMWH, Low Molecular Weight Heparin; LOS, Length of Hospital Stay; LPV/r, Lopinavir/Ritonavir; Lzd, Linezolid; M, Male; MEM, Meropenem; mo., Months; MTM, Metamizol; N/A, Not applicable; NIV, Non-invasive Mechanical Ventilation; PBZ: Pembrolizumab; PCC, Post-COVID Condition; Pred, Prednisolone; RDV, Remdesivir; RXL, Ruxolitinib; TCZ, Tocilizumab; Tec, Teicoplanin; TFV, Tenofovir; TZP, Piperacillin/Tazobactam; Unk, unknown; Vit D, Vitamin D; VOR, Voriconazole.
