# Supplementary material for: Different polarization and functionality of CD4+ T helper subsets in people with post-COVID condition
Source: Front Immunol. 2024 Aug 27;15:1431411. doi: 10.3389/fimmu.2024.1431411 (PMC11385313; doi:10.3389/fimmu.2024.1431411)
Supplement: Supplementary file 2 [file Presentation1.pptx]

## Slide 1
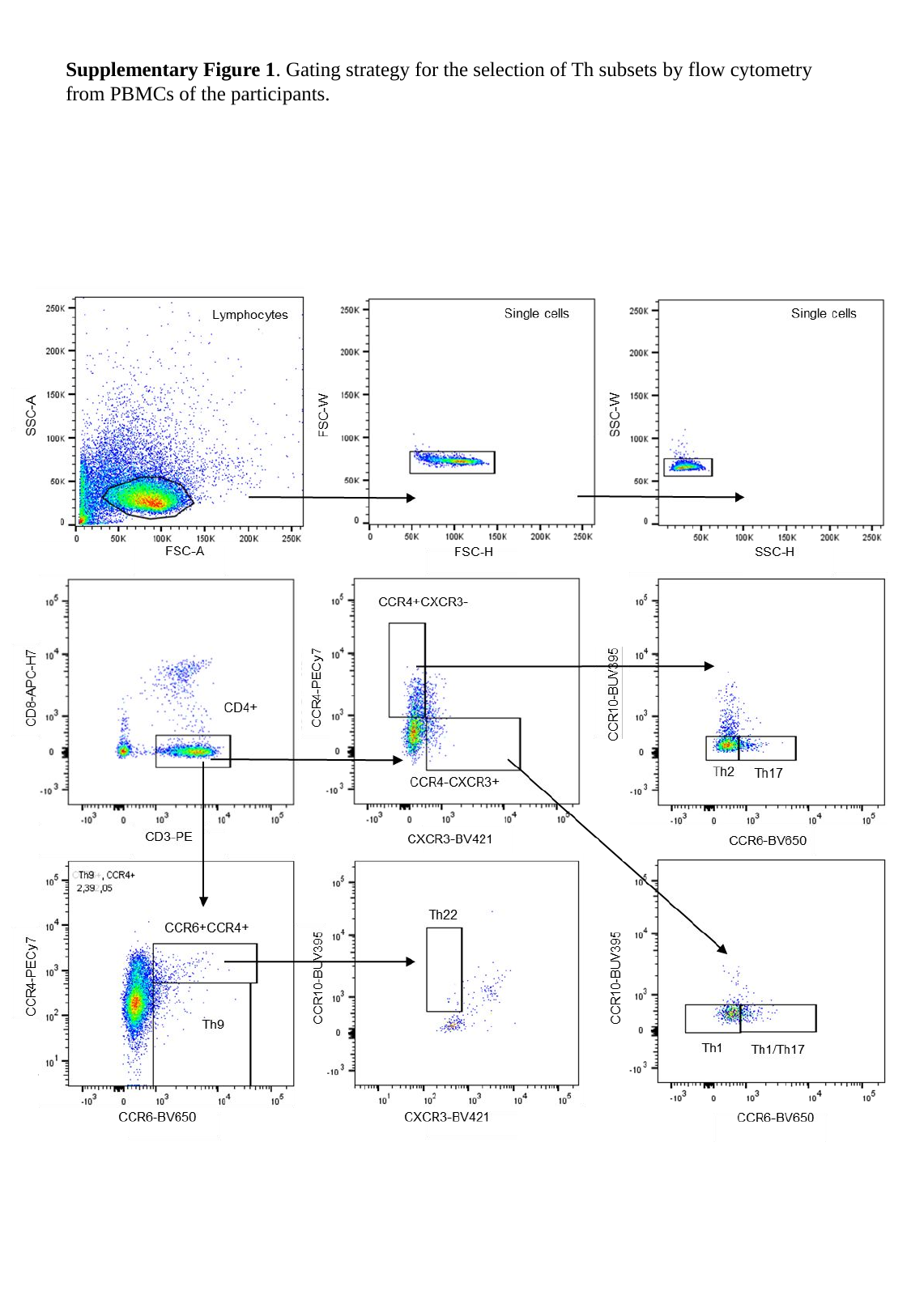

Supplementary Figure 1. Gating strategy for the selection of Th subsets by flow cytometry from PBMCs of the participants.
