# Supplementary material for: Different polarization and functionality of CD4+ T helper subsets in people with post-COVID condition
Source: Front Immunol. 2024 Aug 27;15:1431411. doi: 10.3389/fimmu.2024.1431411 (PMC11385313; doi:10.3389/fimmu.2024.1431411)
Supplement: Supplementary file 3 [file Presentation2.pptx]

## Slide 1
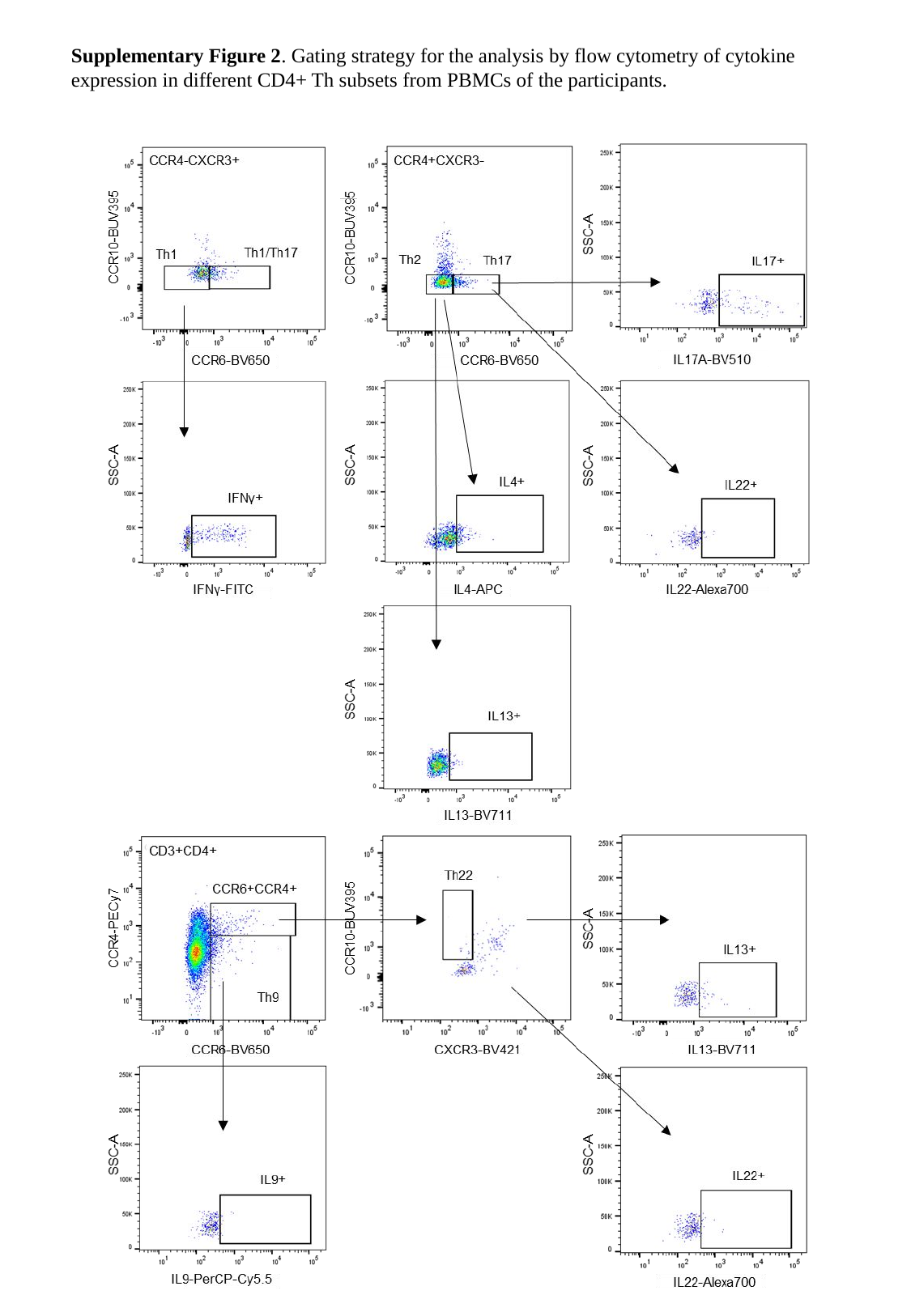

Supplementary Figure 2. Gating strategy for the analysis by flow cytometry of cytokine expression in different CD4+ Th subsets from PBMCs of the participants.
