# Supplementary material for: Different polarization and functionality of CD4+ T helper subsets in people with post-COVID condition
Source: Front Immunol. 2024 Aug 27;15:1431411. doi: 10.3389/fimmu.2024.1431411 (PMC11385313; doi:10.3389/fimmu.2024.1431411)
Supplement: Supplementary file 4 [file Presentation3.pptx]

## Slide 1
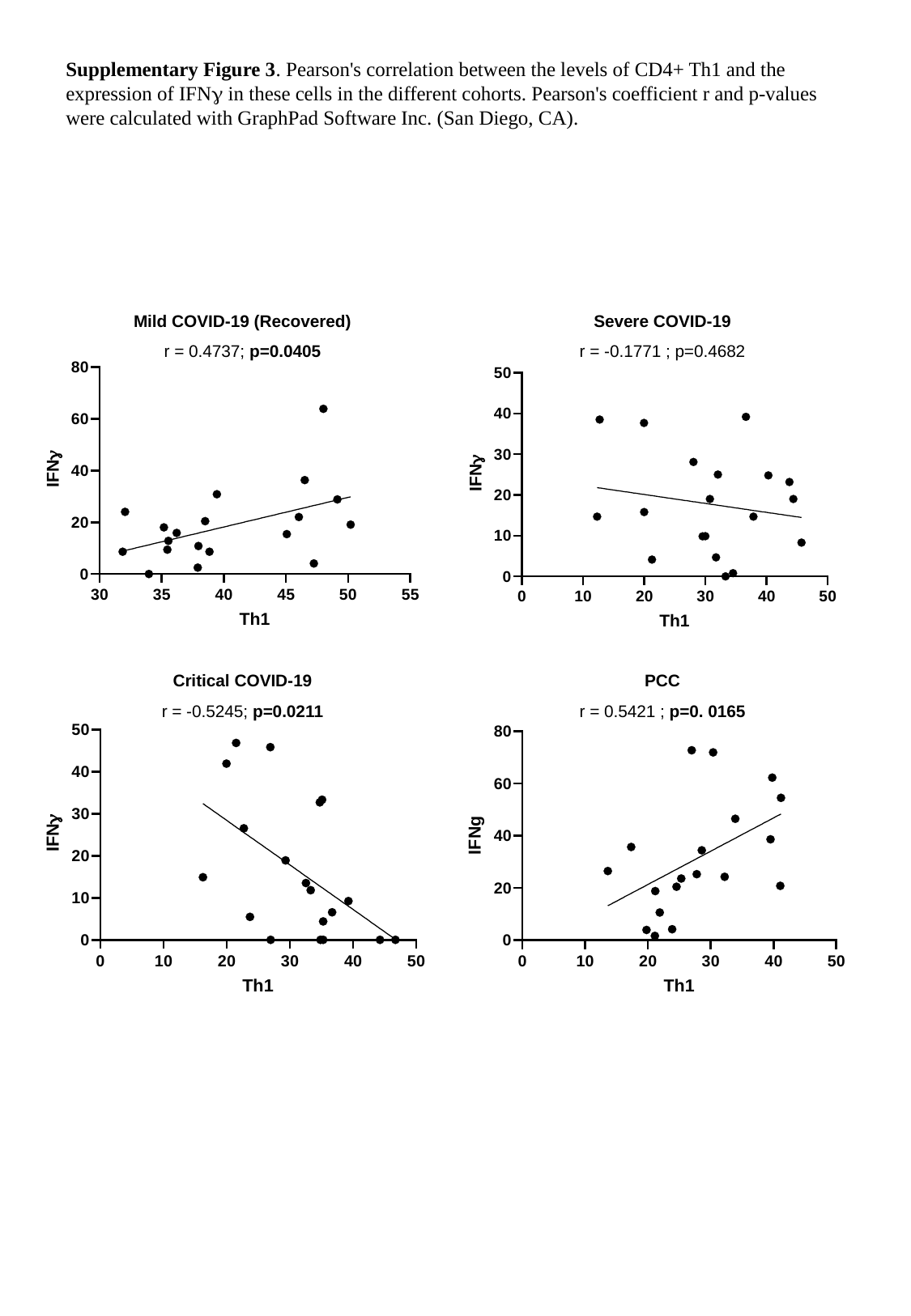

Supplementary Figure 3. Pearson's correlation between the levels of CD4+ Th1 and the expression of IFNg in these cells in the different cohorts. Pearson's coefficient r and p-values were calculated with GraphPad Software Inc. (San Diego, CA).
Mild COVID-19 (Recovered)
r = 0.4737; p=0.0405
Severe COVID-19
r = -0.1771 ; p=0.4682
Critical COVID-19
r = -0.5245; p=0.0211
PCC
r = 0.5421 ; p=0. 0165
