# Supplementary material for: Different polarization and functionality of CD4+ T helper subsets in people with post-COVID condition
Source: Front Immunol. 2024 Aug 27;15:1431411. doi: 10.3389/fimmu.2024.1431411 (PMC11385313; doi:10.3389/fimmu.2024.1431411)
Supplement: Supplementary file 5 [file Presentation4.pptx]

## Slide 1
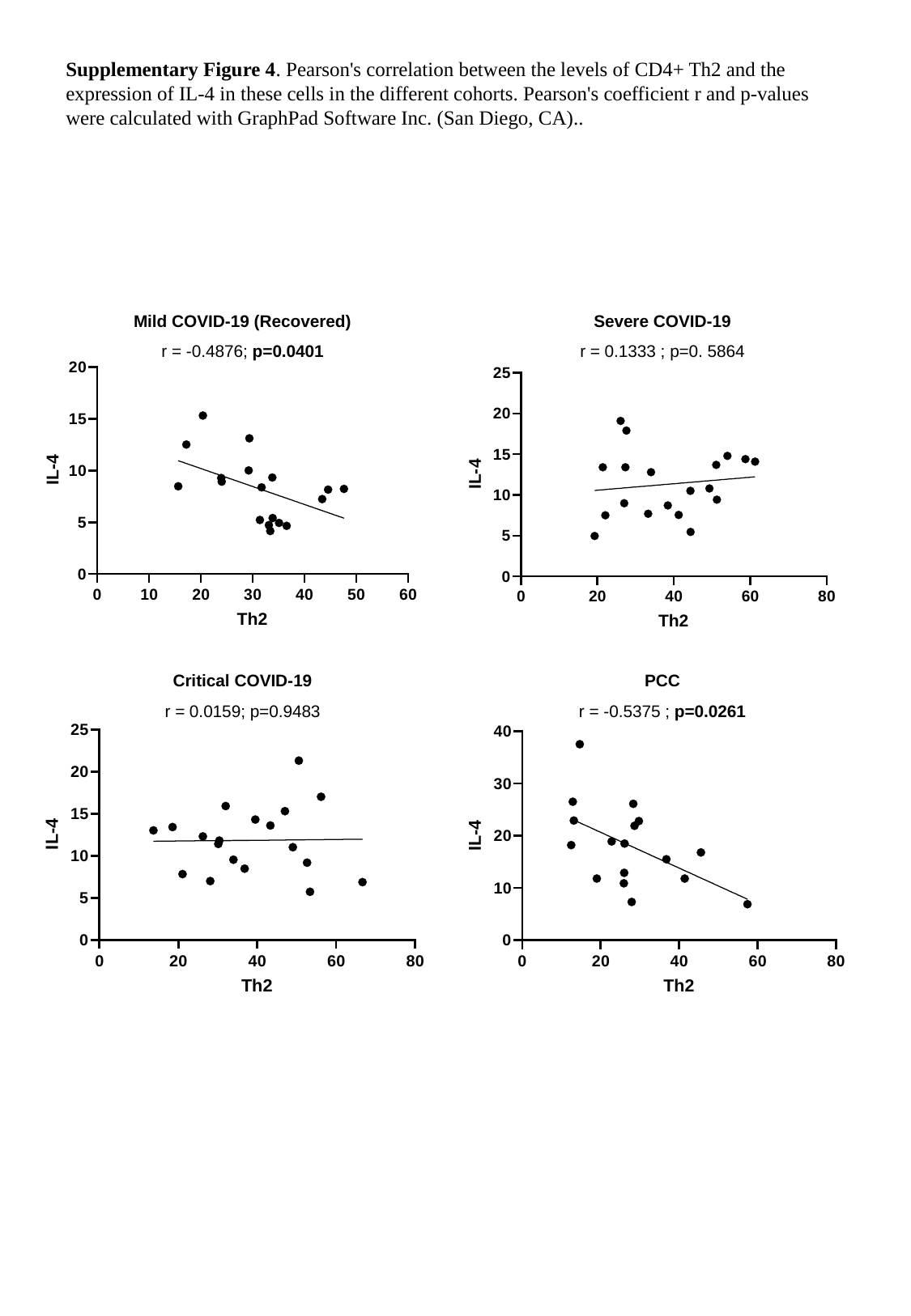

Supplementary Figure 4. Pearson's correlation between the levels of CD4+ Th2 and the expression of IL-4 in these cells in the different cohorts. Pearson's coefficient r and p-values were calculated with GraphPad Software Inc. (San Diego, CA)..
Mild COVID-19 (Recovered)
r = -0.4876; p=0.0401
Severe COVID-19
r = 0.1333 ; p=0. 5864
Critical COVID-19
r = 0.0159; p=0.9483
PCC
r = -0.5375 ; p=0.0261
