# Supplementary material for: Different polarization and functionality of CD4+ T helper subsets in people with post-COVID condition
Source: Front Immunol. 2024 Aug 27;15:1431411. doi: 10.3389/fimmu.2024.1431411 (PMC11385313; doi:10.3389/fimmu.2024.1431411)
Supplement: Supplementary file 7 [file Presentation6.pptx]

## Slide 1
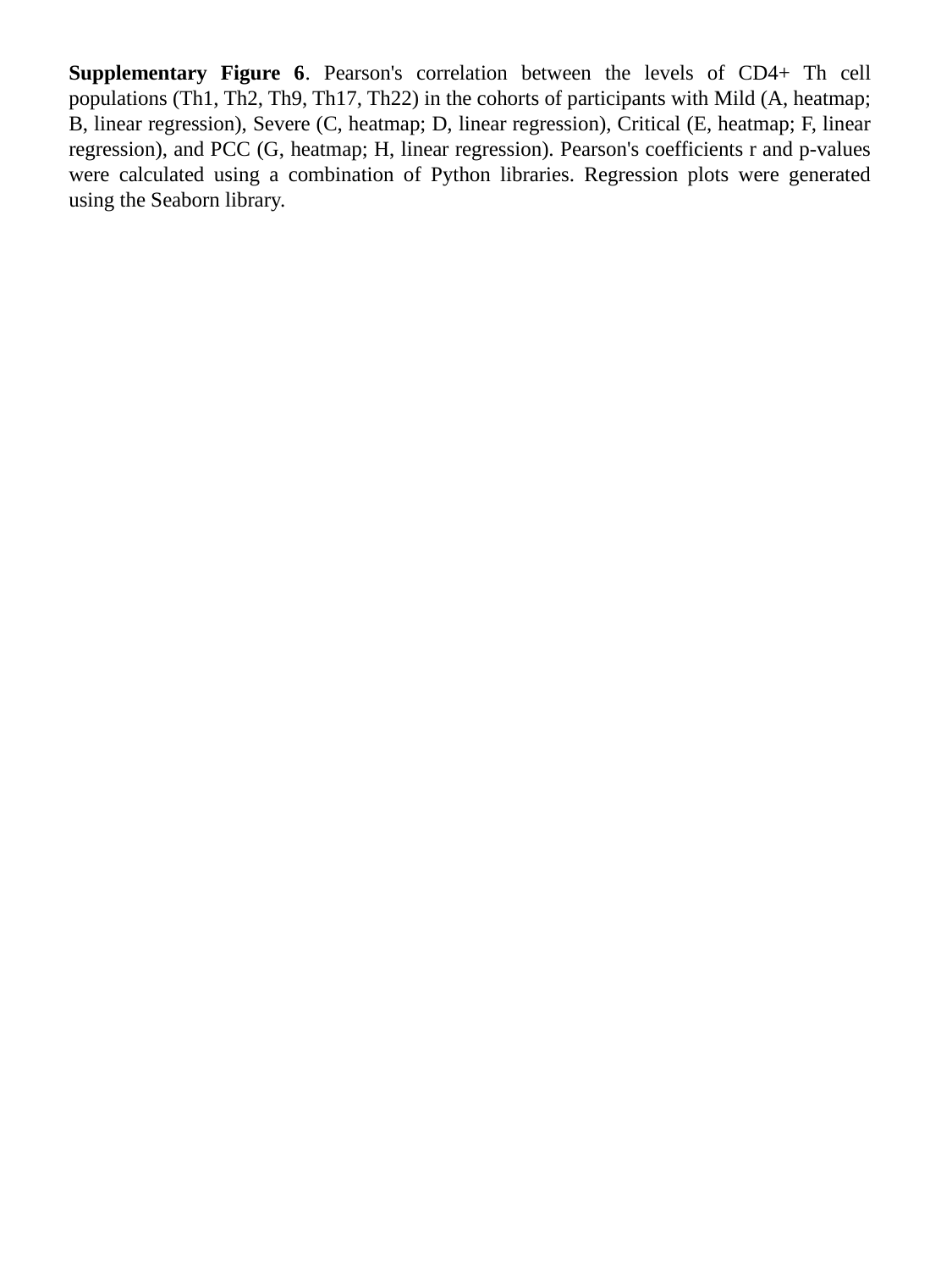

Supplementary Figure 6. Pearson's correlation between the levels of CD4+ Th cell populations (Th1, Th2, Th9, Th17, Th22) in the cohorts of participants with Mild (A, heatmap; B, linear regression), Severe (C, heatmap; D, linear regression), Critical (E, heatmap; F, linear regression), and PCC (G, heatmap; H, linear regression). Pearson's coefficients r and p-values were calculated using a combination of Python libraries. Regression plots were generated using the Seaborn library.

## Slide 2
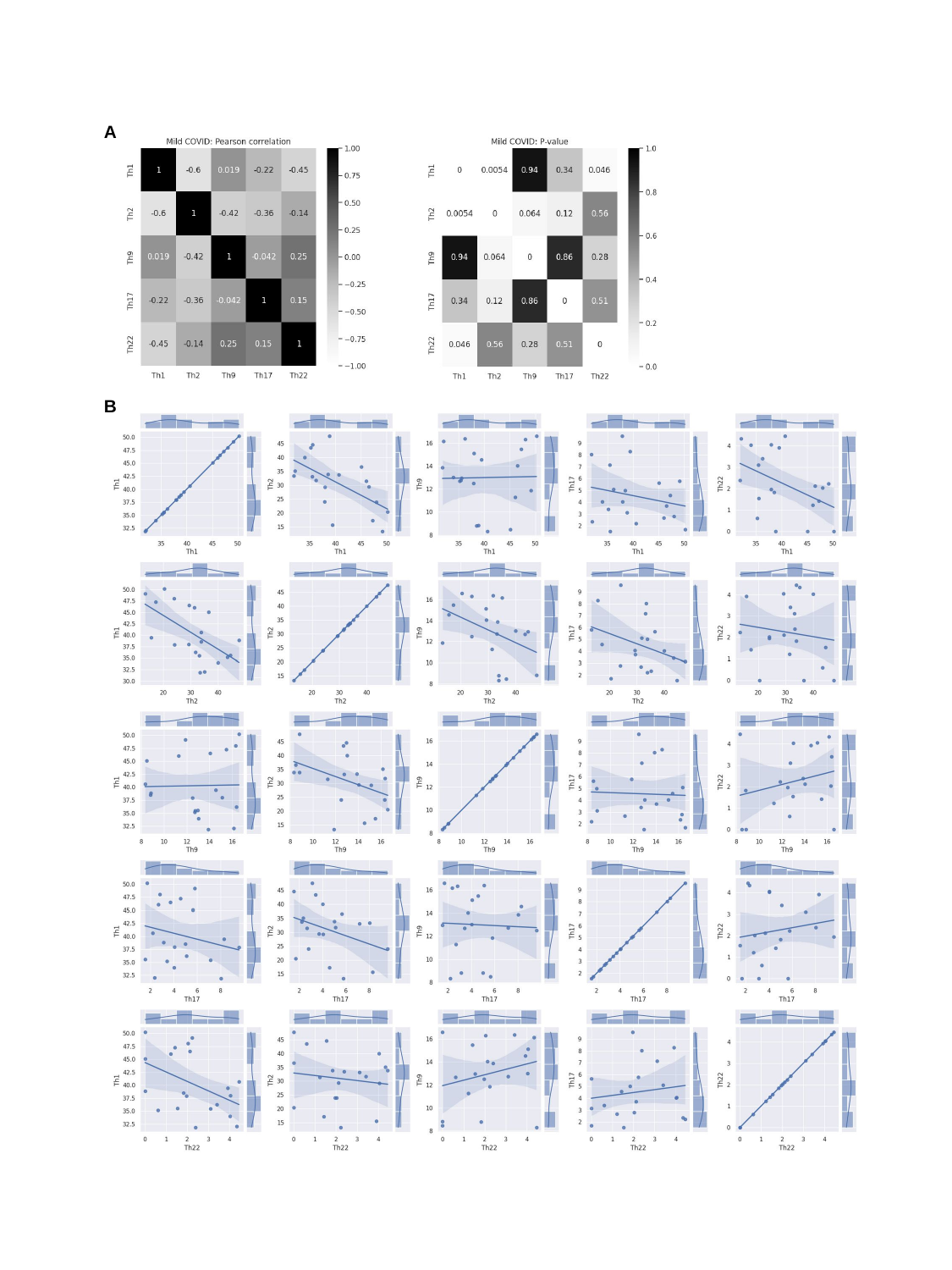

A
B

## Slide 3
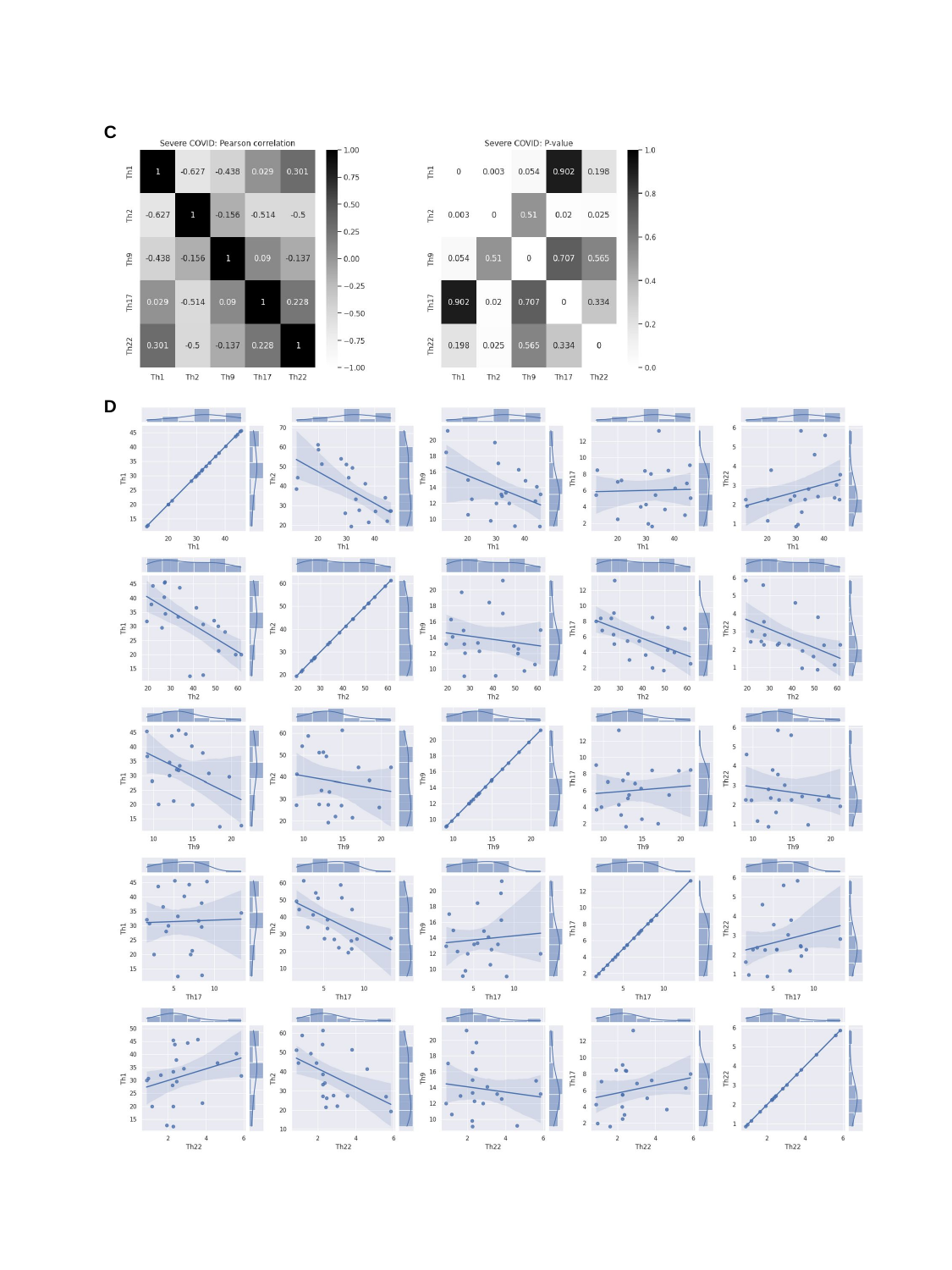

C
D

## Slide 4
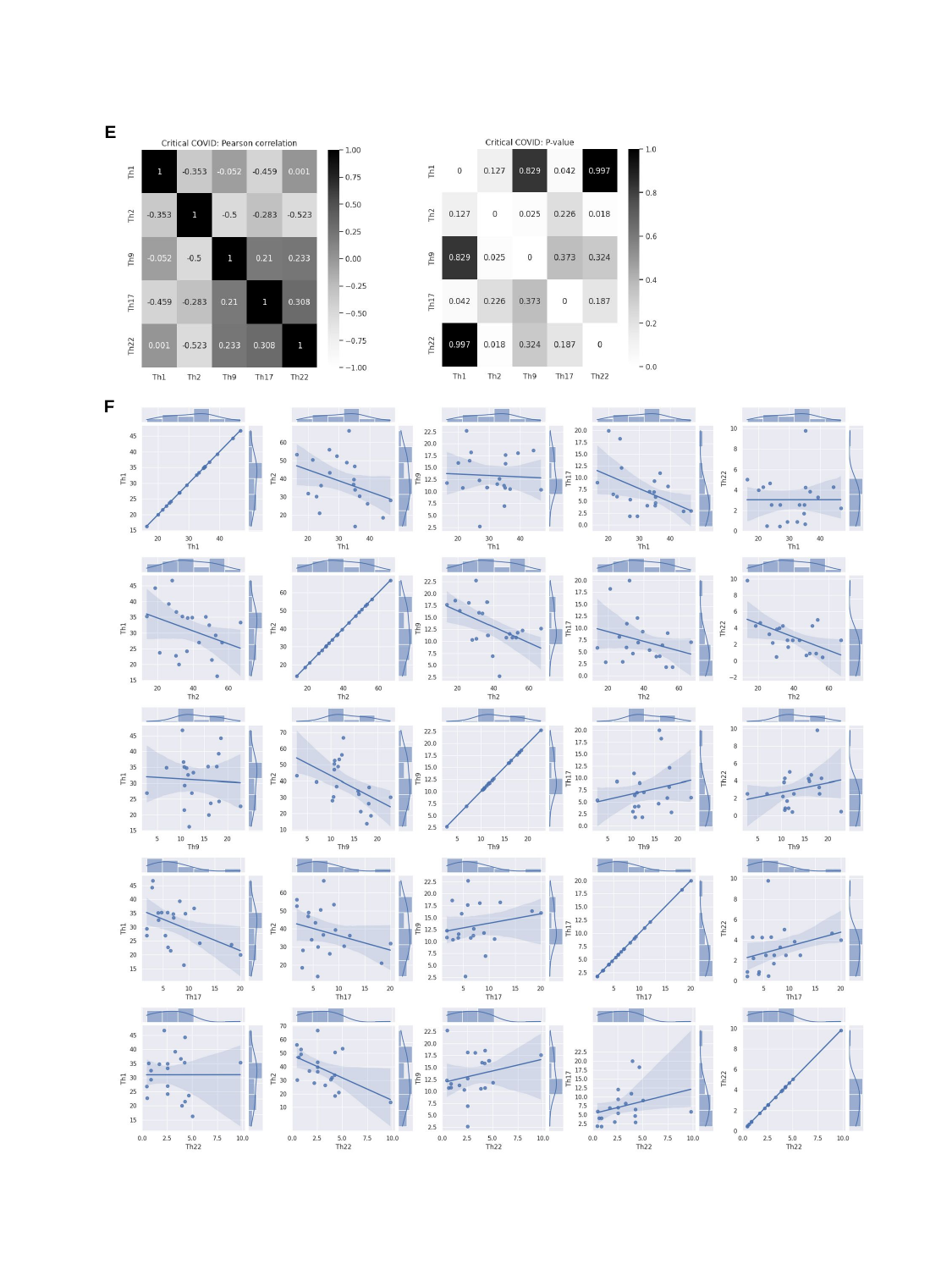

E
F

## Slide 5
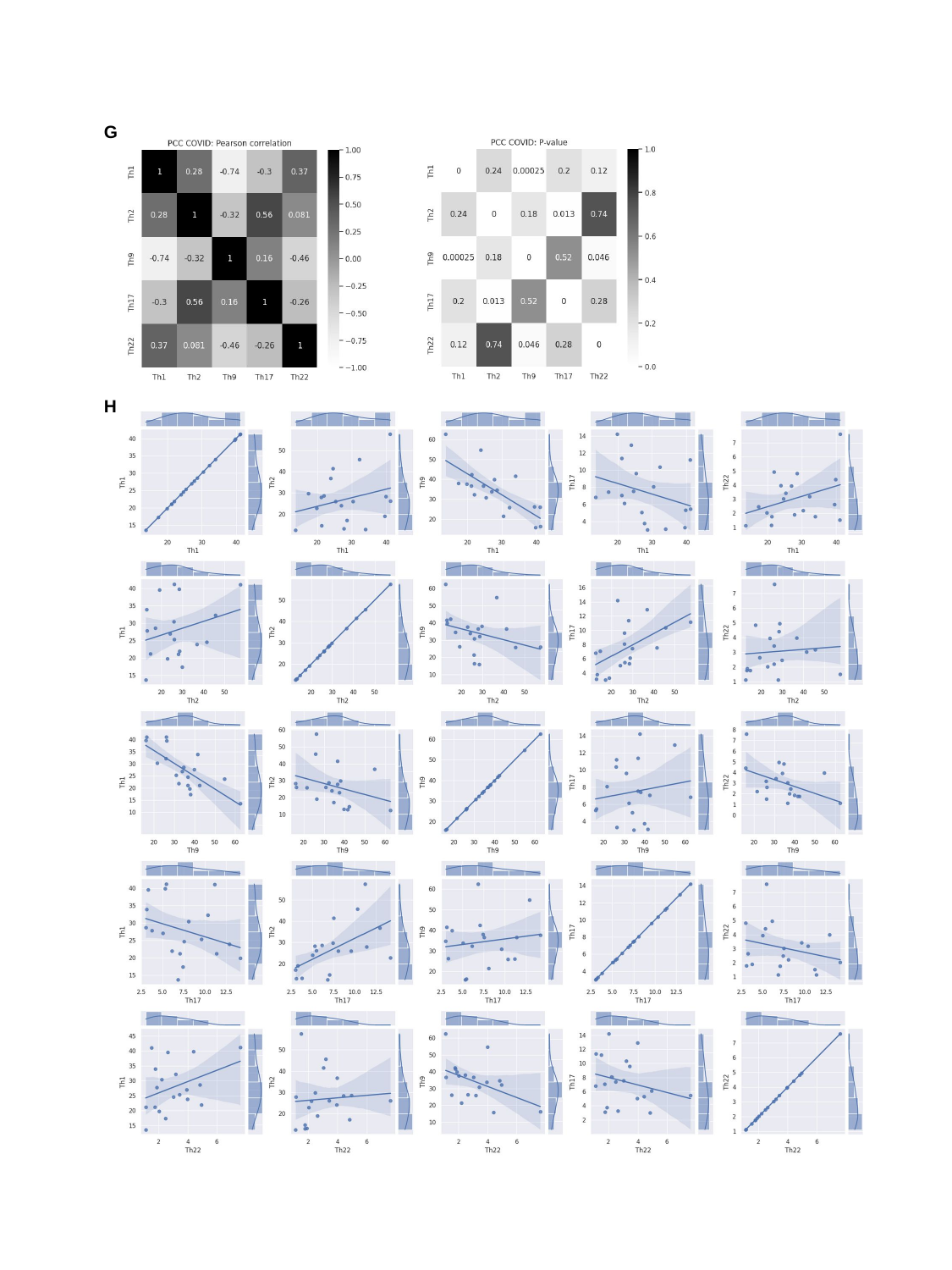

G
H
